# Studying the clinical, radiological, histological, microbiological, and immunological evolution during the different COVID-19 disease stages using minimal invasive autopsy

**DOI:** 10.1038/s41598-022-05186-y

**Published:** 2022-01-25

**Authors:** Valentino D’Onofrio, Lotte Keulen, Annelore Vandendriessche, Jasperina Dubois, Reinoud Cartuyvels, Marie-Elena Vanden Abeele, Judith Fraussen, Patrick Vandormael, Veerle Somers, Ruth Achten, Amélie Dendooven, Ann Driessen, Lukasz Augsburg, Niels Hellings, Martin Lammens, Jan Vanrusselt, Janneke Cox

**Affiliations:** 1 Department of Immunology and Infection, Faculty of Medicine and Life Sciences, Hasselt University, Martelarenlaan 42, 3500 Hasselt, Belgium; 2 Department of Infectious Diseases and Immunity, Jessa Hospital, Stadsomvaart 11, 3500 Hasselt, Belgium; 3 Department of Pathology, Antwerp University Hospital, Edegem, Belgium; 4 Intensive Care and Anesthesiology, Jessa Hospital, Hasselt, Belgium; 5 Clinical Laboratory, Jessa Hospital, Hasselt, Belgium; 6 Department of Geriatrics, Jessa Hospital, Hasselt, Belgium; 7 Department of Pathology, Jessa Hospital, Hasselt, Belgium; 8 Core, University of Antwerp, Wilrijk, Belgium; 9 Department of Pathology, University Hospital Ghent, Ghent, Belgium; 10 Department of Radiology, Jessa Hospital, Hasselt, Belgium

**Keywords:** Immunopathogenesis, Infection, Inflammation, Clinical microbiology, Viral infection, Pathogenesis

## Abstract

The WHO defines different COVID-19 disease stages in which the pathophysiological mechanisms differ. We evaluated the characteristics of these COVID-19 disease stages. Forty-four PCR-confirmed COVID-19 patients were included in a prospective minimal invasive autopsy cohort. Patients were classified into mild-moderate (n = 4), severe-critical (n = 32) and post-acute disease (n = 8) and clinical, radiological, histological, microbiological and immunological data were compared. Classified according to Thoracic Society of America, patients with mild-moderate disease had no typical COVID-19 images on CT-Thorax versus 71.9% with typical images in severe-critical disease and 87.5% in post-acute disease (*P* < 0.001). Diffuse alveolar damage was absent in mild-moderate disease but present in 93.8% and 87.5% of patients with severe-critical and post-acute COVID-19 respectively (*P* = 0.002). Other organs with COVID-19 related histopathological changes were liver and heart. Interferon-γ levels were significantly higher in patients with severe-critical COVID-19 (*P* = 0.046). Anti-SARS CoV-2 IgG was positive in 66%, 40.6% and 87.5% of patients with mild-moderate, severe-critical and post-acute COVID-19 respectively (n.s.). Significant differences in histopathological and immunological characteristics between patients with mild-moderate disease compared to patients with severe-critical disease were found, whereas differences between patients with severe-critical disease and post-acute disease were limited. This emphasizes the need for tailored treatment of COVID-19 patients.

## Introduction

Since the first report in December 2019 of patients infected with severe acute respiratory syndrome corona virus 2 (SARS CoV-2), this virus has travelled the globe. Coronavirus disease (COVID-19) has caused tremendous mortality and morbidity worldwide and continues to do so^1^.

SARS-CoV-2 infection is characterized by different disease stages. After primary infection, patients may either remain asymptomatic, or develop symptoms including fever, fatigue, cough, myalgia, loss of smell or gastro-intestinal complaints, so called mild disease. This can progress to moderate disease, in which the lower respiratory tract becomes infected and can develop further into severe respiratory disease and critical disease leading to respiratory failure, multi-organ failure and death^2^. Moreover, in some patients surviving COVID-19, post-acute COVID-19 syndrome occurs, with persistent and prolonged effects on multiple organ systems^3^. Typically, COVID-19 incubation period is 5 days (range 1–14 days), with progression to severe disease 8 days after disease onset (range 7–14 days) and to critical disease after 16 days (range 12–20 days)^3^. Post-acute COVID-19 syndrome is considered as a disease duration of longer than 4 weeks after symptom onset. Prior to COVID-19 vaccination, approximately 20% of the symptomatic patients evolved towards severe or critical disease and approximately 17–35% of the hospitalized COVID-19 patients required treatment in the intensive care unit (ICU) whereof 20% died^4^. However, these proportions varied depending on infection demographics, thresholds for hospitalization, and availability of ICU beds^1,5^. Post COVID-19 sequelae are reported in 32–87% of patients^6,7^.

The main underlying pathophysiological mechanisms are proposed to vary among different disease stages. Early in the disease course, direct cellular toxicity due to viral replication is believed to be the main driver of pathology, while the progression towards clinically more severe disease is related to a complex dysregulation of the immunological response leading to a hyperinflammatory state including hypercoagulability^8,9^. Moreover, due to prolonged (ICU) admission, patients are at risk for hospital-related complications, including secondary infections and treatment toxicity. The pathophysiology of post-acute COVID-19 syndrome remains unclear, but potentially includes virus-specific changes of cells, inflammatory damage after acute infection and post-critical illness^3^. A clear understanding of the pathophysiology during the different disease stages provides important information for the development of treatment strategies^8^.

We have set up a prospective observational minimal invasive autopsy (MIA) cohort of patients that died with COVID-19. In this paper we report the clinical, radiological, histological, microbiological, and immunological characteristics at different COVID-19 disease stages including patients with post-acute COVID-19.

## Results

### Patient characteristics

**Figure 1:**
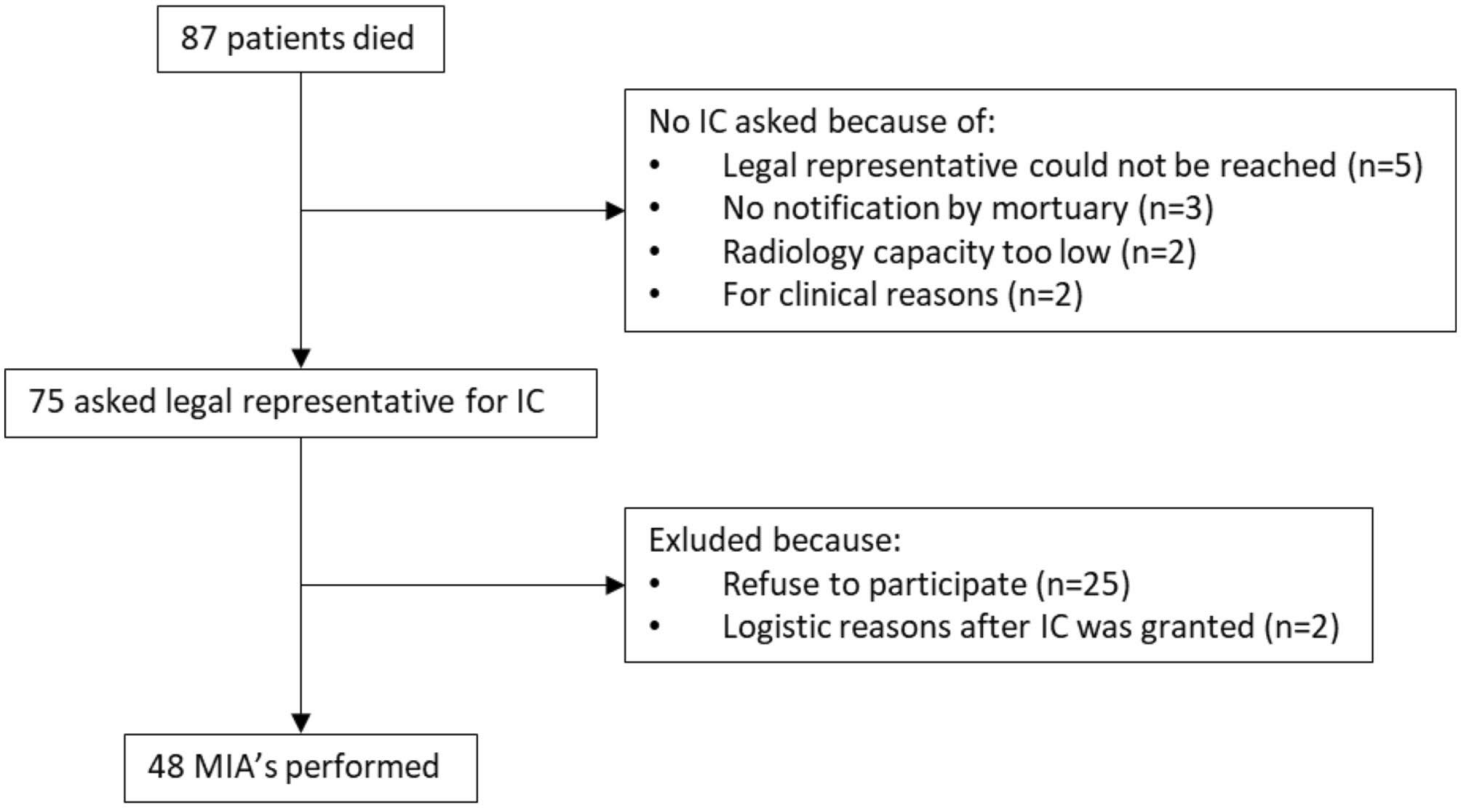
Flowchart of patient inclusions.

A flowchart is provided in Fig. 1. In total, 87 eligible patients died in the Jessa Hospital during the study period, family members of 75 patients were contacted and 48 (64.0%) patients were included, for whom the characteristics are shown in Table 1. The median time from death to MIA was 17h50min (10h54 – 20h58). Forty-four (91.7%) patients were PCR confirmed SARS-CoV-2 positive during illness, and four (8.3%) patients were radiologically confirmed.

**Table 1:** Characteristics of included patients.

	Total (n = 44)	Mild/moderate disease (n = 4)	Severe/critical disease (n = 32)	Post-acute disease (n = 8)
**Age (years, median (IQR)**	82 (73–86)	84 (69–87)	83 (75–88)	75 (69–84)
**Sex (female, n (%))**	19 (43.2)	2 (50.0)	15 (46.9)	2 (25.0)
**Charlson Comorbidity Index (a)**	2 (1–4)	3 (2–5)	2 (1–4)	2 (2–4)
**Duration of symptoms (days, median (IQR))**	12 (7–19)	2 (1.5–2.5)	11 (6–14)	32 (29–46)
**Time between positive PCR and death (days, median (IQR))**	10 (4–19)	4 (2–8)	8 (4–14)	29 (26–39)
**LOS (days, median (IQR))**	11 (4–22)	10 (3–18)	8 (3–19)	23 (23–26)
**Treatment restrictions (b)**				
COVID AA	12 (27.3)	1 (25.0)	6 (18.7)	5 (62.5)
COVID A	10 (22.7)	0 (0.0)	9 (28.1)	1 (12.5)
COVID B	21 (47.7)	2 (50.0)	17 (53.1)	2 (25.0)
COVID C	1 (2.3)	1 (25.0)	0 (0.0)	0 (0.0)
**Respiratory support at death (all)**				
None	2 (4.5)	2 (50.0)	0 (0.0)	0 (0.0)
To a maximum 15L O_2_ on non-rebreathing mask	22 (50.0)	2 (50.0)	18 (56.3)	2 (25.0)
High flow O_2_-therapy	11 (25.0)	0 (0.0)	9 (28.1)	2 (25.0)
Invasive	9 (20.5)	0 (0.0)	5 (15.6)	4 (50.0)
**Dexamethasone treatment**	17 (40.5)	1 (25.0)	14 (45.2)	2 (25.0)
**Remdesivir treatment (c)**	1 (2.3)	0 (0.0)	0 (0.0)	1 (12.5)
**Anticoagulation treatment in the last 2 days before death**	33 (76.7)	1 (25.0)	25 (80.6)	7 (87.5)
**Antibiotic treatment in the last 2 days before death**	20 (45.5)	0 (0.0)	17 (53.1)	3 (37.5)

Values are presented as number (%) except where indicated otherwise. (a): Charlson Comorbidity Index: not age corrected; (b): COVID AAA: no restrictions, including extra-corporal membrane oxygenation (ECMO), COVID AA: allowed invasive oxygen therapy, not ECMO, COVID A: allowed ICU admission, not invasive oxygen therapy, COVID B: maximal treatment without ICU admission, COVID C: comfort therapy; (c): Symptom onset was unknown in 8 patients, disease duration starting from a positive nasopharyngeal PCR was used.

*LOS* length of stay, *IQR* interquartile range.

In none of the four patients with radiologically confirmed COVID-19 we found evidence of SARS-CoV-2 infection. Post-mortem SARS-CoV-2 PCR on lung tissue and serological testing for SARS-CoV-2 antibodies at death were negative for all four patients. Time since symptom onset was 1, 6, 11, and 27 days, respectively. Heart failure (n = 1) and bacterial pneumonia (n = 1) were defined as alternative cause of death (COD) in two patients. The remaining two patients were classified as viral pneumonia of unknown origin as histology showed signs of viral pneumonia, but additional PCR for non-SARS-CoV-2 viral and atypical bacterial respiratory pathogens was negative. As COVID-19 was not confirmed in any of these cases, they were excluded from further analyses.

Four out of 44 (9.1%) patients had mild-moderate disease according to the WHO definition with a median (IQR) disease duration of 2 days (1.5–2.5d). Disease duration was calculated from the time of symptom onset or from a positive PCR test if symptom onset was unknown (Table 1). Most patients had severe-critical disease (n = 32, 72.7%) with a median (IQR) disease duration of 11 days (6-14d) and all had respiratory support at death: supplemental oxygen with a maximum of 15L in 18 (56.3%) patients, high-flow oxygen therapy in nine (28.1%) patients and invasive ventilation in five (15.6%) patients. Eight out of 44 (18.2%) patients had post-acute COVID-19, i.e. a symptom onset longer than 28 days before death. The median (IQR) disease duration was 32 days (29-46d). All eight patients had critical disease during their disease course. At death four patients (50.0%) were on invasive ventilation, two (25.0%) on high-flow oxygen therapy and two (25.0%) on a maximum of 15L O_2_.

### Radiological findings

A detailed description of the CT findings of thorax, abdomen and cerebrum is listed in supplementary Table 1. Five out of 36 (14%) patients showed clinically relevant abnormalities on CT-cerebrum and 8 (18.2%) on the CT-abdomen. There were no significant differences in frequency of abnormalities between disease stage.

Comparing the CT-thorax images, assessed according to Thoracic Society of America classification, revealed significant differences between disease stages. There were no typical images in patients with mild-moderate disease, which was significantly different from patients with severe-critical disease (n = 23, 71.9%) and patients with post-acute disease (n = 7, 87.5%; *P* = 0.003) (Table 2). Although there was no difference in the number of patients with ground glass opacities (GGO) between disease stages, the location and distribution of GGO differed significantly: all (100%) patients with severe-critical or post-acute disease had bilateral GGO, while this was 50% in patients with mild or moderate disease (*P* < 0.001). All patients with mild-moderate disease had a peripheral distribution of GGO while 70.4% and 50.0% of patients with severe-critical disease and post-acute disease, respectively, had a diffuse distribution (*P* = 0.0005). In addition, there were no patients with mild-moderate disease with crazy paving, which was significantly different from other disease stages (*P* = 0.014). Fibrosis was seen only twice, once in mild-moderate disease and once in post-acute disease, and at least the latter was pre-existent.

**Table 2:** Computer tomography findings of the thorax.

	Total (n = 44)	Mild/moderate disease (n = 4)	Severe/critical disease (n = 32)	Post-acute disease (n = 8)
**COVID-19 pneumonia (a)**				
No COVID-19 pneumonia	2 (4.5)	1 (25.0)	0 (0.0)	1 (12.5)
Atypical COVID-19 pneumonia	8 (18.2)	3 (75.0)	5 (15.6)	0 (0.0)
Indeterminate COVID-19 pneumonia	4 (9.1)	0 (0.0)	4 (12.5)	0 (0.0)
Typical COVID-19 pneumonia	30 (68.2)	0 (0.0)	23 (71.9)	7 (87.5)
**Overall severity**				
Mild	7 (15.9)	2 (50.0)	4 (12.5)	1 (12.5)
Moderate	11 (25.0)	2 (50.0)	8 (25.0)	1 (12.5)
Severe	26 (59.1)	0 (0.0)	20 (62.5)	6 (75.0)
**COVID-19 suspected findings**				
Ground glass opacities	37 (84.1)	2 (50.0)	27 (84.4)	8 (100.0)
Crazy Paving	29 (65.9)	0 (0.0)	23 (71.9)	6 (75.0)
Consolidations	39 (88.6)	3 (75.0)	29 (90.6)	7 (87.5)
Organizing pneumonia	1 (2.3)	0 (0.0)	0 (0.0)	1 (12.5)
Fibrosis	2 (4.5)	1 (25.0)	0 (0.0)	1 (12.5)

Values are presented as number (%) except where indicated otherwise; (a) According to Thoracic Society of America.

### Histopathological findings

The most prominent histopathological abnormalities were found in the lungs (Table 3). Other organs with significant changes were liver and heart (Table 4). No COVID-related abnormalities were found in the kidney, spleen, or abdominal fat. All histopathological findings are displayed in supplementary Table 2.

**Table 3:** Main histological changes in the lungs.

	Total (n = 44)	Mild/moderate disease (n = 4)	Severe/critical disease (n = 32)	Post-acute disease (n = 8)
**ARDS/DAD**	37 (84.1)	0 (0.0)	30 (93.8)	7 (87.5)
Early exudative phase	25 (67.8)	0 (0.0)	23 (76.7)	2 (25.0)
Mid proliferative phase	10 (27.0)	0 (0.0)	6 (20.0)	4 (50.0)
Late/organizing fibrotic phase	2 (5.4)	0 (0.0)	1 (3.3)	1(12.5)
**Fibrin deposition/Hyaline membranes**				
None/absent	7 (15.9)	3 (75.0)	3 (9.4)	1 (12.5)
Mild	7 (15.9)	1 (25.0)	4 (12.5)	2 (25.0)
Moderate	18 (40.9)	0 (0.0)	16 (50.0)	2 (25.0)
Severe	12 (27.3)	0 (0.0)	9 (28.1)	3 (37.5)
**Fibrosis**	26 (59.1)	0 (0.0)	21 (65.6)	5 (62.5)
**Lymfocytic infiltrate**				
None/scarse	0 (0.0)	0 (0.0)	0 (0.0)	0 (0.0)
Few	6 (13.6)	2 (50.0)	3 (9.4)	1 (12.5)
Moderate amount	27 (61.4)	2 (50.0)	19 (59.4)	6 (75.0)
Numerous	11 (25.0)	0 (0.0)	10 (31.3)	1 (12.5)
**Macrophages**				
None/scarse	0 (0.0)	0 (0.0)	0 (0.0)	0 (0.0)
Few	8 (18.2)	3 (75.0)	3 (9.4)	2 (25.0)
Moderate amount	23 (52.3)	0 (0.0)	20 (62.5)	3 (27.5)
Numerous	13 (29.5)	1 (25.0)	9 (28.1)	3 (37.5)
**Thrombi**	1 (2.3)	0 (0.0)	1 (3.2)	0 (0.0)
**Pneumocyte atypia**	32 (72.3)	1 (25.0)	26 (81.3)	6 (75.0)
**Megakaryocytes**				
None	19 (43.2)	1 (25.0)	13 (41.9)	5 (62.5)
One	5 (11.4)	1 (25.0)	3 (9.4)	1 (12.5)
Two or more	20 (45.5)	2 (50.0)	16 (50.0)	2 (25.0)
**Vasculitis**	3 (6.8)	0 (0.0)	1 (3.2)	2 (25.0)

Values are presented as number (%) except where indicated otherwise.

**Table 4:** Main histological changes in the heart and liver.

Liver	Total (n = 44)	Mild/moderate disease (n = 4)	Severe/critical disease (n = 32)	Post-acute disease (n = 8)
**Sinus dilatation**				
Absent or mild	16 (36.4)	1 (25.0)	9 (28.1)	6 (75.0)
Moderate	20 (45.5)	3 (75.0)	16 (50.0)	1 (12.5)
Severe	8 (18.2)	0 (0.0)	7 (21.9)	1(12.5)
**Lobular inflammation**				
None/absent	33 (75.0)	3 (75.0)	27 (84.4)	3 (37.5)
Mild	9 (20.5)	1 (25.0)	4 (12.5)	4 (50.0)
Moderate	0 (0.0)	0 (0.0)	0 (0.0)	0 (0.0)
Severe with necrosis	2 (4.5)	0 (0.0)	1 (3.1)	1 (12.5)

Heart	Total (n = 42)	Mild/moderate disease (n = 4)	Severe/critical disease (n = 30)	Post-acute disease (n = 8)
**Fibrosis**				
None/absent	20 (47.6)	3 (75.0)	15 (50.0)	2 (25.0)
Mild	13 (31.0)	1 (25.0)	9 (30.0)	3 (37.5)
Moderate	4 (9.5)	0 (0.0)	2 (6.7)	2 (25.0)
Severe	5 (11.9)	0 (0.0)	4 (13.3)	1 (12.5)
**Acute myocarditis**	1 (2.4)	0 (0.0)	0 (0.0)	1 (12.5)

#### Lung

Diffuse alveolar damage (DAD) was absent in patients with mild-moderate disease, while present in 93.8% and 87.5% of patients with severe-critical or post-acute COVID-19 respectively (*P* = 0.002). Moreover, we mainly saw early exudative DAD in severe-critical COVID-19, where mid proliferative phase was most prevalent in post-acute disease, while late organizing DAD was virtually absent in both groups. Hyaline membranes were absent in patients with mild-moderate disease, while present in 81.3% of patients with severe-critical COVID-19 and 62.5% of patients with post-acute COVID-19 (*P* = 0.019). Pneumocyte atypia was more prevalent in severe-critical disease (81.3%) and post-acute disease (75%) than in mild-moderate disease (25.0%) (n.s.). Acute neutrophilic inflammation compatible with bacterial pneumonia was equally present in all groups. Overall, we rarely saw vasculitis (3/44) and thrombi (1/44). The composition of inflammatory infiltrates differed mainly between the mild-moderate group and the other groups. Moderate to numerous macrophages were present in 90.6% of patients with severe-critical disease and in 75% with post-acute disease, compared to 25.0% of patients with mild-moderate disease (*P* = 0.042) and the presence of lymphocytes was most prominent in patients with severe-critical (90.6%) and post-acute (87.5%) disease compared to patients with mild-moderate disease (50.0%) (*P* = 0.024). The lymphocytic infiltrates consisted mainly of CD3 + T-lymphocytes. In only two patients a discordance between lymphocytic infiltrate and CD3 expression was found (4.5%) where the lymphocytic infiltrates were scored as moderate, while CD3 + cells were scored as scarce. Microscopic fibrosis (deposition of collagen and accumulation of fibroblasts) was seen in none of the patients with mild-moderate disease, while this was present in 65.6% of patients with severe-critical disease and in 62.5% of patients with post-acute COVID (n.s.).

#### Liver

The most important finding in all liver biopsies was prominent dilation of the sinusoids, without significant differences between disease stages. The presence of lobular inflammation was, in contrast, limited to a few patients and mostly mild or moderate.

#### Heart

Biopsies of the heart were obtained in 42 patients. Histological findings were similar in all three groups. Nineteen patients presented with moderate to severe fibrosis of cardiac tissue (21.4%), indicating underlying long-standing cardiovascular disease. Acute myocarditis was found in only one patient (2.3%) with post-acute COVID-19 disease. In three patients, circulating neutrophils were found in cardiac capillaries, but not enough evidence of interstitial inflammation was seen (7.1%).

### Microbiological findings

Cultures on post-mortem lung tissue revealed 22 pathogens in 17/44 (38.6%) patients, mainly gram-negative bacteria (15/22, 68.1%) (Supplementary Table 3). Three (75.0%) patients with mild-moderate disease, 11 (34.4%) patients with severe-critical disease and three (37.5%) patients with post-acute COVID-19 had positive cultures with relevant pathogens (n.s.). When correlated to clinical, radiological, and histological findings, 13 (29.5%) patients had a bacterial or fungal infection as the COD or contributing diagnosis, two (50%) with mild-moderate disease, nine (28%) with severe-critical disease and two (25%) with post-acute COVID-19.

### Immunological findings

**Figure 2:**
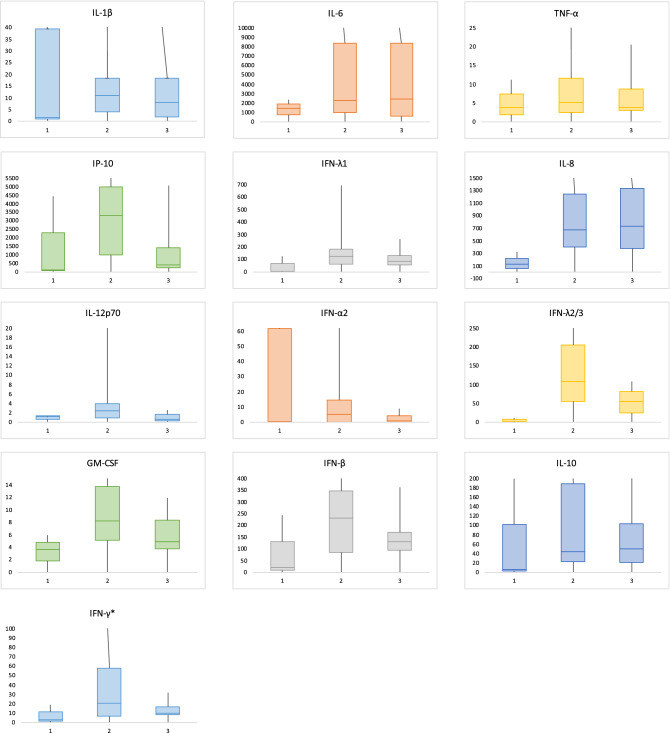
Postmortem cytokine levels in plasma per disease stage. 1: mild-moderate disease; 2: severe-critical disease; 3: post-acute disease.

Median concentrations of 13 cytokines in plasma of deceased patients collected post-mortem are shown per disease stage in Fig. 2. Interferon-γ (IFNγ) levels were significantly higher in patients with severe-critical disease (*P* = 0.046). Although there were no other significant differences in cytokine levels, a trend was observed for higher levels of interleukin 6 (IL-6), IFNγ-induced protein 10 (IP-10) and granulocyte macrophage colony stimulating factor (GM-CSF) and lower levels of interferon-γ (IFNγ) in patients with severe-critical disease. Dexamethasone treatment was not associated with cytokine levels.

None of the included patients were vaccinated against COVID-19. Overall, 31/43 (72%, 1 missing sample) patients were seropositive for anti-SARS-CoV-2 antibodies of the IgM isotype, while 22/43 (51%) were positive for IgG. Two (66%) patients with mild-moderate disease, 22 (68.8%) patients with severe-critical disease and seven (87.5%) patients with post-acute disease had anti-SARS-CoV-2 IgM antibodies, and two (66%), 13 (40.6%) and seven (87.5%) had anti-SARS-CoV-2 IgG antibodies, respectively.

Of the 21 IgG negative patients, 17 (81%) had a disease duration of ≤ 12 days. The four IgG negative patients with a disease duration of > 12 days could be considered immunosuppressed with underlying B-cell lymphoma (n = 1), hemodialysis (n = 1), amyloidosis (n = 1), and non-Hodgkin lymphoma with immunosuppressive medication, including rituximab and high dosage steroids (n = 1).

Comparison of anti-SARS-CoV-2 IgG levels according to the NIBSC standard, to a cutoff value associated with 6-month protection from infection^10^ showed that only 12/43 (28%) had sufficiently high anti-SARS-CoV-2 IgG levels.

When correlating cytokine levels with antibody positivity, no significant differences were found, but a trend towards higher IP-10 levels in patients with negative IgM antibodies was observed (*P* = 0.054).

### Cause of death

**Figure 3:**
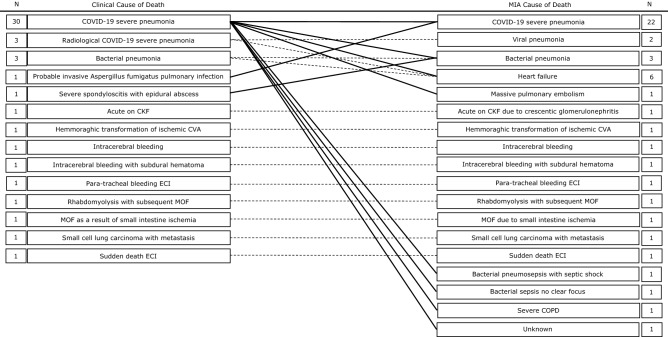
Clinical causes of death and changes to MIA cause of death.

The COD and all contributing diagnoses are shown in Fig. 3 and supplementary Table 4. The number of patients with severe-critical disease that died of COVID-19 pneumonia (59.4%) was greater than that of patients with mild-moderate disease (0%) and patients with post-acute disease (37.5%), although this difference was not significant (*P* = 0.06).

In patients with mild-moderate disease, the COD was heart failure (n = 1, 25%), small cell lung carcinoma (n = 1, 25%), multi-organ failure due to small intestine ischemia (n = 1, 25%), and hemorrhagic and semi-recent ischemic cerebrovascular accident (n = 1, 25%). The COD in patients with severe-critical disease was COVID-19 severe pneumonia in 19 (59.4%) patients, heart failure in four (12.5%), bacterial pneumonia in two (6.3%), intracerebral bleeding in two (6.3%), and sepsis, massive pulmonary embolism, severe COPD and multi-organ failure due to rhabdomyolysis, each in one (3.1%) patient. The COD in one patient was unknown. The COD in patients with post-acute disease were COVID-19 severe pneumonia (n = 3, 37.5%), heart failure (n = 1, 12.5%), sepsis (n = 1, 12.5%), sudden death of unknown origin (n = 1, 12.5%), crescentic glomerulonephritis (n = 1, 12.5%), and paratracheal bleeding (n = 1, 12.5%).

## Discussion

This prospective observational MIA cohort in SARS-CoV-2 infected patients describes the clinical, radiological, histopathological, microbiological, and immunological differences between different disease stages. Patients with mild-moderate disease had fewer abnormalities on CT, did not show DAD in lung biopsies and had a less pronounced cytokine response compared to patients with severe-critical disease. However, differences between patients with severe-critical disease and post-acute disease were rather limited. Radiology showed equally high prevalence of crazy paving and bilateral consolidation. Histologically, DAD was present equally in both groups with > 60% of patients with post-acute COVID-19 in early exudative or mid proliferative stage. Immunologically, no significant differences in cytokine responses, except IFNy, were seen.

In none of the four radiologically confirmed COVID-19 patients we found post-mortem confirmation of SARS-CoV-2 infection, despite elaborate investigations. This emphasises the need to reconsider the diagnosis of COVID-19 if the molecular confirmation in lacking, and to optimize the diagnostic strategy for both COVID-19 and alternative diagnoses.

Furthermore, despite having a disease onset of > 28 days, most patients with post-acute COVID-19 had a radiological, histological, and immunological profile of acute respiratory distress syndrome. One may postulate that patients included in this cohort i.e., hospitalized patients that died, had an aberrant disease course that did not show a dampening of the acute lung damage and failed to progress to a more chronic phase^11,12^. Even in patients with the longest disease duration (> 32 days) and in those treated with dexamethasone, no trend toward chronic disease was observed. This confirms the idea of acute inflammation as an important cause of mortality^13,14^. Although groups are small, this may have important implications for treatment of these patients, as they may benefit, even at such a long disease duration, from (prolonged) immunomodulating treatments like those given to patients with severe-critical disease.

An interesting finding was the difference in composition of the inflammatory infiltrate, with macrophages still being present in a relatively high percentage in relation to the degree of fibrosis in both severe-critical and post-acute disease. Macrophages play an important and dual pro- and anti-inflammatory role in ARDS. Literature suggests that the M1 type is present in earlier phases, releasing pro-inflammatory cytokines, while the M2 type is present in later phases to eliminate apoptotic cells, thereby possibly contributing to fibrosis^15^. Histopathological fibrosis was seen in a high percentage of patients with severe-critical and post-acute disease. It must be noted that not all patients with fibrosis showed typical DAD features. Some patients showed mild to moderate fibrosis thought to be pre-existent, without any other signs of DAD.

Next, megakaryocytes were easily found in a large proportion of patients. It has been reported that an increased number of pulmonary megakaryocytes, responsible for production of platelets, can be seen in lung tissue of COVID-19 patients with DAD. This is thought to underline the relation with embolic/thrombotic events reported in COVID-19 patients^16^. Nevertheless, a low number of thrombi and vasculitis were seen in this cohort, much lower than commonly reported in the literature^17,18^. One possible reason is that we performed MIA instead of full autopsies and obtained tissue cores of approximately 5X30mm, which has a higher chance of ‘sampling error’, especially to catch relatively large structures like thrombi and larger vessels.

We found profound abnormalities in the lungs and liver, but no distinctive COVID-related findings were found in the other organs that were investigated. In other autopsy series, various abnormalities were described in virtually all organs. However, in these studies, complete autopsies were performed allowing for more extensive tissue sampling of more organs and a higher yield of tissue per organ^12,19^. We collected per protocol lung biopsies from radiological abnormal and normal tissue, however disease involvement of all lobes was very frequent, and “radiologically normal” lung tissue was limited. This was confirmed histologically as biopsies labelled “radiologically normal” had only in 3/44 (6.8%) cases no or few abnormalities.

No significant differences in cytokine responses, except for IFNy were found between disease stages. IP-10, IL-6, and GM-CSF levels tended to be higher in patients with severe-critical disease compared to mild-moderate disease. These are pro-inflammatory cytokines, typically seen during cytokine storm and related to hyperinflammation-induced severe disease. Histopathological findings in the lung could support this hypothesis: lymphocytes and plasma cells were more prominent in patients with severe-critical disease.

A trend towards decreased cytokine levels was seen in post-acute COVID-19 and this could point to an increased immune response followed by a start to return to normal however they did not return to levels as low as during mild-moderate disease. None of the patients with post-acute COVID-19 had leukopenia shortly before death, and therefore this observation cannot be explained by immune exhaustion. Of course, these interpretations should be assessed with caution because of small groups, differences were not significant, and cytokines were measured in plasma which is not necessarily representative of findings in the lung. Furthermore, this is a cohort of the most ill patients. We do not know if these findings truly reflect COVID-19 or reflect other complications during their disease.

IFNγ levels tended to be higher in patients with mild-moderate disease. IFNγ is a type I interferon and has been shown to be an important anti-viral response cytokine related to COVID-19 severity. An impaired IFN type I response was previously reported to be present in patients that developed critical disease^20^. Moreover, patients with inborn errors in type I IFN are at risk of developing life-threatening COVID-19^21^. In addition, the observed trend for higher IP-10 levels in IgM negative patients points toward an impaired immune response. Our findings could indicate that mild disease patients but not patients with severe-critical or post-acute disease, had an adequate antiviral response and therefore did not develop typical COVID-19 pneumonia and may have died of other causes than the virus or hyperinflammation.

Almost 30% of our patients had a bacterial pneumonia, which is higher than the 14% co- or sur-infection rate in mixed wards/ICU settings reported in the literature^22^. This may be explained by selection bias, i.e., the most severely ill patients were included in this cohort. Nevertheless, restricted antibiotic use should be propagated from an antimicrobial stewardship perspective.

Our study has limitations. Although MIA can be a good alternative to conventional autopsies in terms of revealing clinically undiagnosed conditions, tissue sample size is relatively small, which limits a good overview of all architectural abnormalities that might be present leading to sampling erro^23^. As mentioned earlier, this may explain the low number of thrombi found in our study. Also, certain crucial organs like the brain have not been sampled. Moreover, during (ICU) admission, patients generally develop multiple problems, leading to organ damage and changes in inflammatory response. It is therefore impossible to disentangle what abnormalities have been caused by SARS CoV-2 and what not. The inclusion of a control group of SARS-CoV-2 negative patients would have helped overcome this.

In conclusion, we found that patients that die during different COVID-19 disease stages show certain distinct clinical, radiological, histological, and immunological features. Patients with mild-moderate disease showed relatively few pathological abnormalities with a probable adequate immune response and did not die of but with COVID-19. Patients with severe-critical disease showed extensive pulmonary abnormalities, typically reflecting an overreactive and distorted immune response, and mostly died because of COVID-19 pneumonia. Lastly, patients with post-acute disease, despite some trends towards a dampened immunological response, mostly have similar clinical, radiological, and histopathological features compared to severe-critical disease and might therefore benefit from (prolonged) immunomodulating treatment.

The study of pathophysiological changes during different disease stages remains important to elucidate the mechanisms of this new disease. The distinct features during different disease stages show that a tailored and personal management of COVID-19 patients is necessary.

## Methods

### Study design and patients

**Figure 4:**
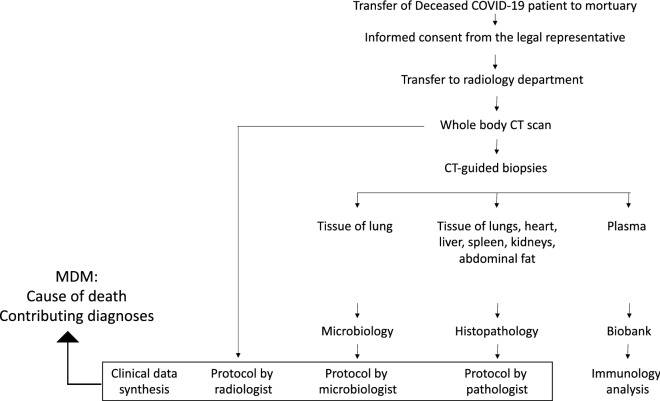
Study Procedure. *CT* computer tomography, *MDM* multidisciplinary meeting.

This study is a prospective cohort. A subset of our cohort has been described in a previous publication^23^. All patients that died during hospitalization with polymerase chain reaction (PCR) or radiologically confirmed COVID-19 were eligible for inclusion. Radiologically confirmed COVID-19 was defined as a person in whom PCR testing for COVID-19 is negative, but in whom the diagnosis is made on the basis of a suggestive clinical presentation and a compatible CT-scan^24^. Inclusion took place during working hours (9 am to 5 pm), 7 days per week from 15th April 2020 until 24th December 2020. MIAs were performed on the day of study inclusion (Fig. 4). A maximum of 2 MIAs were performed per day.

### Informed consent and ethical approval

Formally, no informed consent (IC) is needed for autopsy to be performed in tertiary and academic centres in Belgium. Nevertheless, we sought IC from the legal representative. Because of visiting restrictions in the hospital during the COVID-19 outbreak, most often the legal representative was contacted by phone by a study team member, who explained the study and asked oral IC. If consent was granted, the study information sheet was sent by registered mail. All patients were included after oral informed consent from the legal representative. The study received ethical approval from the Ethics Committee of Jessa hospital and Hasselt University (20.36-infecti20.05). All procedures performed were in accordance with the ethical standards of the institutional and national research committee and with the 1964 Helsinki Declaration and its later amendments or comparable ethical standards. Clinicaltrials.gov identifier: NCT 04,366,882.

### MIA procedure

A full body CT-scan was performed in a 128-slice CT scanner (Somatom go.top; Siemens Healthcare, Erlangen, Germany) with the body in supine position. Volumetric scans were obtained from the vertex to the symphysis pubis at 120 kV with variable mAs, without intravenous or intra-arterial contrast. Image reconstruction with a soft tissue algorithm provided 3 mm slices, which were viewed on standard window settings for soft tissue, lung and bone.

Tissue biopsies were taken with the ‘cutting needle alone’ technique, using a 14G biopsy needle (Bard ® Mission™ Disposable Core Biopsy Instrument; C.R. Bard, Inc., Tempe, AZ, USA). Four lung biopsies were collected for microbiological examination, at least 4 biopsies of each lung (2 biopsies radiologically normal and 2 biopsies radiologically abnormal) for histopathological examination and at least 2 biopsies from heart, kidney, liver, spleen, and abdominal fat for histopathological examination. Blood (15 ml) was collected from the aorta in serum, lithium heparin and citrate tubes (Vacuette, Greiner-Bio, Vilvoorde, Belgium). Additional tissue and blood were collected for biobank storage at University Biobank Limburg (UBiLim) at -80 °C.

### Microbiology

Samples for microbiological examination were cultured in a level 3 safety lab. Lung tissue was inoculated on standard culture media for bacteria, yeasts and fungi and microorganisms were identified by Matrix-assisted laser desorption ionization–time of flight mass spectrometry. For all radiologically confirmed COVID-19 patients, we performed an in-house respiratory PCR panel on lung tissue on Quantstudio 7 flex (ThermoFisher) for the simultaneous detection of 23 respiratory pathogens, including SARS-CoV-2, influenza, RSV, adenovirus, enterovirus, human metapneumovirus, parainfluenza, rhinovirus, *Bordetella holmesii*, *Bordetella pertussis*, *Bordetella parapertussis*, *Chlamydophila pneumoniae*, *Legionella pneumophila,* and *Mycoplasma pneumoniae*.

### Histopathology

Samples for histopathological examination were stored in 10% neutral buffered formalin for 72 h and embedded in paraffin. Haematoxylin and eosin staining was performed on all specimens. At least one lung biopsy per patient was stained with both CD3 immunohistochemical and elastin histochemical stains to assess the severity of lung disease. Additional stains were performed (CD4, CD8, Congo Red, elastine von Gieson, other) when deemed indicated by the pathologist.

### Immunological analyses

The LegendPlex Human Anti-Virus Response Panel (13-plex) (740,390, BioLegend) was used according to the manufacturer’s instructions with minor adjustments. The assay was carried out in V-bottom 96-well plates and serum (12.5 µL) was thawed and diluted twofold with assay buffer before testing. Standards, mixed beads, detection antibodies and streptavidin-PE were prepared according to the manufacturer’s instructions and 12.5µL of each reagent was used. All serum samples were tested in duplicate. Data were collected using a LSRFortessa flow cytometer (BD Biosciences) and analysed using the LEGENDplex™ Data Analysis Software Suite (BioLegend). Means of detection limits of duplicate tests were calculated and were used as a reference for cytokine levels under the detection limit. IgM and IgG antibodies against the S1 subunit of the SARS-CoV-2 S-protein were detected in serum or plasma samples using enzyme linked immunosorbent assays (ELISA) (IgM, Beijing Wantai Biological; IgG, Euroimmun), according to the manufacturer’s instructions. Samples were considered seropositive according to the cut-off of the respective ELISA kits. IgG and IgM antibody levels were quantified by linear interpolation using serial dilutions of a positive plasma sample, which was later converted to arbitrary units (AU)/mL using the Anti-SARS-CoV-2 Antibody Diagnostic Calibrant (20/162) from the National Institute for Biological Standards and Control (NIBSC). Samples were measured twice independently, and the coefficient of variation of the average AU/ml was lower than 30% for seropositive samples.

### Data collection

The deceased patients’ electronic medical file was assessed by at least one clinician who summarized medical history, discharge letters, file notes, and pre-mortem laboratory and radiological assessments. The post-mortem CT-scans were assessed by at least one radiologist following a standardized protocol. Pneumonias were classified as typical, indeterminate, atypical and normal according to the Radiological Society of North America classification^25^. The post-mortem lung cultures were assessed by a microbiologist. Culture results were scored as pathogen or contaminant/colonization based on identification and quantity. The histology slides were reviewed by four independent pathologists who subsequently discussed the findings of each organ and provided one conclusive finding based on consensus. The stages of DAD were scored according to hyaline membranes, inflammatory infiltrate, extent of fibrosis and divided into three groups: early exudative phase, mid proliferative phase, and late/organizing phase^26^. Inflammation was scored as being absent, mild, moderate, or severe. The composition of inflammatory infiltrates was assessed by scoring certain inflammatory cells as being absent, scarce, moderate, or numerous in number.

### Multidisciplinary meetings

Each patient was discussed during multidisciplinary meetings with at least one clinician, one radiologist, one pathologist, and one microbiologist. During the meeting a summary of the medical chart, the post-mortem CT-images, the microbiological and histopathological findings were presented. Then, the participants discussed the results and defined the COD and contributing diagnoses based on consensus.

### Statistical analyses

Patients were classified according to the WHO definitions of mild-moderate or severe-critical COVID disease at the moment of death^27^. Patients with a symptom onset of 28 days or more, were classified as post-acute COVID-19. If the moment of symptom onset was unavailable, the moment of positive PCR-testing was used. Comparison was made between mild-moderate disease, severe-critical disease, and post-acute COVID-19. Data are presented as number (percentage) for categorical data or median (IQR) for continuous data. Mann Whitney U test (continuous) or Chi-square test (categorical data) were used. A p-value < 0.05 was considered significant. All analyses were done using SPSS (version 25, IBM).

## Supplementary Information

Supplementary Tables.

## Data Availability

The datasets used and analysed during the current study are available from the corresponding author on reasonable request. The data are not publicly available due to them containing information that could compromise research participant privacy/consent.
