# Supplementary material for: Studying the clinical, radiological, histological, microbiological, and immunological evolution during the different COVID-19 disease stages using minimal invasive autopsy
Source: Sci Rep. 2022 Jan 25;12:1360. doi: 10.1038/s41598-022-05186-y (PMC8789771; doi:10.1038/s41598-022-05186-y)
Supplement: Supplementary file 1 — Supplementary Tables. [file 41598_2022_5186_MOESM1_ESM.docx]

**The evolution of COVID-19: a minimal invasive autopsy cohort studying in depth the different COVID-19 disease stages**

Valentino D’Onofrio^1,2,*^, Lotte Keulen^3^, Annelore Vandendriessche^3^, Jasperina Dubois^4^, Reinoud Cartuyvels^5^, Marie-Elena Vanden Abeele^6^, Judith Fraussen^1^, Patrick Vandormael^1^, Veerle Somers^1^, Ruth Achten^7,8^, Amélie Dendooven^3,8,9^, Ann Driessen^3,8^, Lukasz Augsburg^10^, Niels Hellings^1^, Martin Lammens^3,8^, Jan Vanrusselt^10^, Janneke Cox^1,2,*^

**Supplementary information**

**Supplementary Table 1.** Major radiological findings

|  | **Total (n=44)** | **Mil/Moderate disease (n=4)** | **Severe/critical disease (n=32)** | **Post-acute disease (n=8)** |
| --- | --- | --- | --- | --- |
| **Cerebrum*** |  |  |  |  |
| No abnormality | 29/34 (85.3) | 2/3 (66.7) | 21/23 (91.3) | 6/8 (75.0) |
| Abnormalities | 5/34 (14.7) | 1/3 (33.3) | 2/23 (8.7) | 2/8 (25.0) |
| Bleeding | 4/5 (80.0) | 1 (100.0) | 2 (100.0) | 1 (50.0) |
| Air | 2/5 (40.0) | 0 (0.0) | 1 (50.0) | 2 (100.0) |
| Edema | 2/5 (40/0) | 0 (0.0) | 2 (100.0) | 0 (0.0) |
| Loss of gray and white tissue differentiation | 2/5 (40.0) | 0 (0.0) | 2 (100.0) | 0 (0.0) |
| **Thorax** |  |  |  |  |
| **COVID-19 suspected findings** |  |  |  |  |
| Ground glass opacities | 37 (84.1) | 2 (50.0) | 27 (84.4) | 8 (100.0) |
| Location |  |  |  |  |
| Unilateral | 1/37 (2.7) | 1 (50.0) | 0 (0.0) | 0 (0.0) |
| Bilateral | 36/37 (97.3) | 1 (50.0) | 27 (100.0) | 8 (100.0) |
| Distribution |  |  |  |  |
| No specific distribution | 6/37 (16.2) | 0 (0.0) | 5 (18.5) | 1 (12.5) |
| Perihilar | 1/37 (2.7) | 0 (0.0) | 1 (3.7) | 0 (0.0) |
| Peripheral | 4/37 (10.8) | 2 (100.0) | 1 (3.7) | 1 (12.5) |
| Multifocal | 3/37 (8.1) | 0 (0.0) | 1 (3.7) | 2 (25.0) |
| Diffuse | 23/37 (62.2) | 0 (0.0) | 19 (70.4) | 4 (50.0) |
| Shape |  |  |  |  |
| Rounded | 1/37 (2.7) | 0 (0.0) | 0 (0.0) | 1 (12.5) |
| Non-rounded | 36/37 (97.3) | 2 (100.0) | 27 (100.0) | 7 (87.5) |
| Number of lobes involved (median, range) | 5 (2-5) | 2.5 (2-3) | 5 (3-5) | 5 (5-5) |
| Severity |  |  |  |  |
| Mild | 11/37 (29.7) | 2 (100.0) | 6 (22.2) | 3 (37.5) |
| Moderate | 5/37 (13.5) | 0 (0.0) | 4 (14.8) | 1 (12.5) |
| Severe | 21/37 (56.8) | 0 (0.0) | 17 (63.0) | 4 (50.0) |
| Crazy Paving | 29 (65.9) | 0 (0.0) | 23 (71.9) | 6 (75.0) |
| Location |  |  |  |  |
| Unilateral | 0/29 (0.0) | 0 (0.0) | 0 (0.0) | 0 (0.0) |
| Bilateral | 29/29 (100.0) | 0 (0.0) | 23 (100.0) | 6 (100.0) |
| Distribution |  |  |  |  |
| No specific distribution | 5/29 (17.2) | 0 (0.0) | 5 (21.7) | 0 (0.0) |
| Perihilar | 0/29 (0.0) | 0 (0.0) | 0 (0.0) | 0 (0.0) |
| Peripheral | 0/29 (0.0) | 0 (0.0) | 0 (0.0) | 0 (0.0) |
| Multifocal | 3/29 (10.3) | 0 (0.0) | 1 (4.3) | 2 (33.3) |
| Diffuse | 21/29 (72.4) | 0 (0.0) | 17 (73.9) | 4 (66.7) |
| Number of lobes involved (median, Range) | 5 (5-5) | NA | 5 (5-5) | 5 (5-5) |
| Severity |  |  |  |  |
| Mild | 4/29 (13.8) | 0 (0.0) | 3 (13.0) | 1 (16.7) |
| Moderate | 5/29 (17.2) | 0 (0.0) | 4 (17.4) | 1 (16.7) |
| Severe | 20/29 (69.0) | 0 (0.0) | 16 (69.6) | 4 (66.7) |
| Consolidations | 39 (88.6) | 3 (75.0) | 29 (90.6) | 7 (87.5) |
| Location |  |  |  |  |
| Unilateral | 8/29 (20.5) | 2 (66.7) | 5 (17.2) | 1 (14.3) |
| Bilateral | 31/39(79.5) | 1 (33.3) | 24 (82.8) | 6 (85.7) |
| Distribution |  |  |  |  |
| No specific distribution | 13/39 (33.3) | 2 (66.7) | 9 (31.0) | 2 (28.6) |
| Perihilar | 0/39 (0.0) | 0 (0.0) | 0 (0.0) | 0 (0.0) |
| Peripheral | 1/39 (2.6) | 0 (0.0) | 1 (3.4) | 0 (0.0) |
| Multifocal | 6/39 (15/4) | 0 (0.0) | 4 (13.8) | 2 (28.6) |
| Diffuse | 14/39 (35.9) | 1 (33.3) | 11 (37.9) | 2 (28.6) |
| Lobar | 5/39 (12.8) | 0 (0.0) | 4 (13.8) | 1 (14.3) |
| Number of lobes involved (median, Range) | 5 (1-5) | 3 (1-3) | 5 (1-5) | 5 (1-5) |
| Severity |  |  |  |  |
| Mild | 14/39 (35.9) | 2 (66.7) | 10 (34.5) | 2 (28.6) |
| Moderate | 9/39 (23.1) | 1 (33.3) | 7 (24.1) | 1 (14.3) |
| Severe | 16/39 (41.0) | 0 (0.0) | 12 (41.4) | 4 (57.1) |
| Organizing pneumonia | 1 (2.3) | 0 (0.0) | 0 (0.0) | 1 (12.5) |
| Fibrosis | 2 (4.5) | 1 (25.0) | 0 (0.0) | 1 (12.5) |
| **Non-typical COVID-19 findings** |  |  |  |  |
| Pleural fluid | 31 (70.5) | 2 (50.0) | 22 (68.7) | 7 (87.5) |
| Lymph nodes | 21 (47.7) | 3 (75.0) | 15 (46.9) | 3 (37.5) |
| Cavitation | 1 (2.3) | 0 (0.0) | 1 (3.1) | 0 (0.0) |
| **COVID-19 pneumonia according to Thoracic Society of America** |  |  |  |  |
| No COVID-19 pneumonia | 2 (4.5) | 1 (25.0) | 0 (0.0) | 1 (12.5) |
| Atypical COVID-19 pneumonia | 8 (18.2) | 3 (75.0) | 5 (15.6) | 0 (0.0) |
| Indeterminate COVID-19 pneumonia | 4 (9.1) | 0 (0.0) | 4 (12.5) | 0 (0.0) |
| Typical COVID-19 pneumonia | 30 (68.2) | 0 (0.0) | 23 (71.9) | 7 (87.5) |
| **Overall severity** |  |  |  |  |
| Mild | 7 (15.9) | 2 (50.0) | 4 (12.5) | 1 (12.5) |
| Moderate | 11 (25.0) | 2 (50.0) | 8 (25.0) | 1 (12.5) |
| Severe | 26 (59.1) | 0 (0.0) | 20 (62.5) | 6 (75.0) |
| **Abdomen** |  |  |  |  |
| No abnormality | 36 (81.8) | 2 (50.0) | 28 (87.5) | 6 (81.8) |
| Abnormalities | 8 (18.2) | 2 (50.0) | 4 (12.5) | 2 (25.0) |
| Pancreatitis | 2 (25.0) | 1 (50.0) | 1 (25.0) | 0 (0.0) |
| Perforation | 1 (12.5) | 1 (50.0) | 0 (0.0) | 0 (0.0) |
| Ascites | 1 (12.5) | 0 (0.0) | 1 (25.0) | 0 (0.0) |
| Hematoma | 1 (12.5) | 0 (0.0) | 0 (0.0) | 1 (50.0) |
| Spondylodiscitis | 1 (12.5) | 0 (0.0) | 1 (25.0) | 0 (0.0) |
| Diffuse severe atherosclerosis | 2 (25.0) | 0 (0.0) | 1 (25.0) | 1 (50.0) |

Legend: * Not scanned in 12 patients

**Supplementary Table 2.** Major histopathological findings

|  | **Total**  **(n=44)** | **Mil/Moderate disease (n=4)** | **Severe/critical disease (n=32)** | **Post-acute disease (n=8)** |
| --- | --- | --- | --- | --- |
| **Left Lung** |  |  |  |  |
| ARDS/DAD | 31 (72.1) | 0 (0.0) | 26 (83.9) | 5 (62.5) |
| Early exudative phase | 21 (67.7) | 0 (0.0) | 18 (69.2) | 3 (60.0) |
| Mid proliferative phase | 9 (29.0) | 0 (0.0) | 7 (26.9) | 2 (40.0) |
| Late/organizing fibrotic phase | 1 (3.2) | 0 (0.0) | 1 (3.8) | 0 (0.0) |
| Fibrin/Hyalin (n=1 not able to score) |  |  |  |  |
| None/absent | 11 (25.6) | 3 (75.0) | 5 (16.1) | 3 (37.5) |
| Mild | 4 (9.3) | 1 (25.0) | 3 (9.7) | 0 (0.0) |
| Moderate | 18 (41.9) | 0 (0.0) | 16 (51.6) | 2 (25.0) |
| Severe | 10 (23.3) | 0 (0.0) | 7 (22.6) | 3 (37.5) |
| Fibrosis (n=1 not able to score) | 22 (51.2) | 0 (0.0) | 17 (54.8) | 5 (62.5) |
| Acute inflammation |  |  |  |  |
| None/absent | 14 (31.8) | 1 (25.0) | 11 (34.4) | 2 (25.0) |
| Mild | 20 (45.5) | 2 (50.0) | 14 (43.8) | 4 (50.0) |
| Moderate | 6 (13.6) | 0 (0.0) | 4 (12.5) | 2 (25.0) |
| Severe | 4 (9.1) | 1 (25.0) | 3 (9.4) | 0 (0.0) |
| Chronic inflammation |  |  |  |  |
| None/absent | 1 (2.3) | 1 (25.0) | 0 (0.0) | 0 (0.0) |
| Mild | 10 (22.7) | 2 (50.0) | 7 (21.9) | 1 (12.5) |
| Moderate | 23 (52.3) | 0 (0.0) | 19 (59.4) | 4 (50.0) |
| Severe | 10 (22.7) | 1 (25.0) | 6 (18.8) | 3 (37.5) |
| CD3 (n=20 missing) |  |  |  |  |
| None/scarse | 0 (0.0) | 0 (0.0) | 0 (0.0) | 0 (0.0) |
| Few | 7 (29.2) | 0 (0.0) | 6 (37.5) | 1 (16.7) |
| Moderate amount | 15 (62.5) | 2 (100.0) | 8 (50.0) | 5 (83.3) |
| Numerous | 2 (8.3) | 0 (0.0) | 2 (12.5) | 0 (0.0) |
| Lymfocytic infiltrate |  |  |  |  |
| None/scarse | 0 (0.0) | 0 (0.0) | 0 (0.0) | 0 (0.0) |
| Few | 11 (25.0) | 2 (50.0) | 7 (21.9) | 2 (25.0) |
| Moderate amount | 27 (61.4) | 2 (50.0) | 20 (62.5) | 5 (62.5) |
| Numerous | 6 (13.6) | 0 (0.0) | 5 (15.6) | 1 (12.5) |
| Plasma cells |  |  |  |  |
| None/scarse | 36 (81.8) | 3 (75.0) | 26 (81.3) | 7 (87.5) |
| Few | 6 (13.6) | 1 (25.0) | 4 (12.5) | 1 (12.5) |
| Moderate amount | 1 (2.3) | 0 (0.0) | 1 (3.1) | 0 (0.0) |
| Numerous | 1 (2.3) | 0 (0.0) | 1 (3.1) | 0 (0.0) |
| Eosinophils |  |  |  |  |
| None/scarse | 27 (61.4) | 3 (75.0) | 20 (62.5) | 4 (50.0) |
| Few | 15 (34.1) | 1 (25.0) | 10 (31.3) | 4 (50.0) |
| Moderate amount | 2 (4.5) | 0 (0.0) | 2 (6.3) | 0 (0.0) |
| Numerous | 0 (0.0) | 0 (0.0) | 0 (0.0) | 0 (0.0) |
| Macrophages |  |  |  |  |
| None/scarse | 2 (4.5) | 0 (0.0) | 1 (3.1) | 1 (12.5) |
| Few | 10 (22.7) | 3 (75.0) | 6 (18.8) | 1 (12.5) |
| Moderate amount | 21 (47.7) | 0 (0.0) | 18 (56.3) | 3 (37.5) |
| Numerous | 11 (25.0) | 1 (25.0) | 7 (21.9) | 3 (37.5) |
| Thrombi (n=1 missing) | 1 (2.3) | 0 (0.0) | 1 (3.2) | 0 (0.0) |
| Pneumocyte atypia | 26 (65.9) | 1 (25.0) | 22 (68.8) | 6 (75.0) |
| Findings contributing to death |  |  |  |  |
| No pathological findings | 0 (0.0) | 0 (0.0) | 0 (0.0) | 0 (0.0) |
| Findings unlikely to contribute to death | 2 (4.5) | 1 (25.0) | 1 (3.1) | 0 (0.0) |
| Findings possibly contributing to death | 5 (11.4) | 0 (0.0) | 3 (9.4) | 2 (25.0) |
| Findings probably contributing to death | 9 (20.5) | 2 (50.0) | 7 (21.9) | 0 (0.0) |
| Findings likely to contribute to death | 28 (63.6) | 1 (25.0) | 21 (65.6) | 6 (75.0) |
| Megakaryocytes (n=1 missing) |  |  |  |  |
| None | 24 (55.8) | 1 (25.0) | 17 (54.8) | 6 (75.0) |
| One | 2 (4.7) | 1 (25.0) | 1 (3.2) | 0 (0.0) |
| Two or more | 17 (39.5) | 2 (50.0) | 13 (41.9) | 2 (25.0) |
| Vasculitits | 1 (2.3) | 0 (0.0) | 0 (0.0) | 1 (12.5) |
| CT-normal vs. CT-abnormal |  |  |  |  |
| Different | 11 (25.0) | 2 (50.0) | 6 (18.8) | 3 (37.5) |
| More or less the same | 33 (75.0) | 2 (50.0) | 26 (81.3) | 5 (62.5) |
| **Right Lung** |  |  |  |  |
| ARDS/DAD (n=2 missing) | 29 (69.0) | 0 (0.0) | 23 (76.7) | 6 (75.0) |
| Early exudative phase | 19 (65.5) | 0 (0.0) | 17 (73.9) | 2 (33.3) |
| Mid proliferative phase | 9 (31.0) | 0 (0.0) | 6 (26.1) | 3 (50.0) |
| Late/organizing fibrotic phase | 1 (3.4) | 0 (0.0) | 0 (0.0) | 1 (16.7) |
| Fibrin/Hyalin (n=2 missing) |  |  |  |  |
| None/absent | 11 (26.2) | 4 (100.0) | 5 (16.7) | 2 (25.0) |
| Mild | 7 (16.7) | 0 (0.0) | 5 (16.7) | 2 (25.0) |
| Moderate | 14 (33.3) | 0 (0.0) | 13 (43.3) | 1 (12.5) |
| Severe | 10 (23.8) | 0 (0.0) | 7 (23.3) | 3 (37.5) |
| Fibrosis (n=2 missing) | 21 (50.0) | 0 (0.0) | 16 (53.3) | 5 (62.5) |
| Acute inflammation (n=2 missing) |  |  |  |  |
| None/absent | 14 (33.3) | 2 (50.0) | 9 (30.0) | 3 (37.5) |
| Mild | 19 (45.2) | 2 (50.0) | 13 (43.3) | 4 (50.0) |
| Moderate | 3 (7.1) | 0 (0.0) | 3 (10.0) | 0 (0.0) |
| Severe | 6 (14.3) | 0 (0.0) | 5 (16.7) | 1 (12.5) |
| Chronic inflammation (n=2 missing) |  |  |  |  |
| None/absent | 0 (0.0) | 0 (0.0) | 0 (0.0) | 0 (0.0) |
| Mild | 8 (19.0) | 3 (75.0) | 3 (10.0) | 2 (25.0) |
| Moderate | 22 (52.4) | 0 (0.0) | 19 (63.3) | 22 (52.4) |
| Severe | 12 (28.6) | 1 (25.0) | 8 (26.7) | 3 (37.5) |
| CD3 (n= 27 missing) |  |  |  |  |
| None/scarse | 0 (0.0) | 0 (0.0) | 0 (0.0) | 0 (0.0) |
| Few | 5 (29.4) | 2 (100.0) | 2 (15.4) | 1 (50.0) |
| Moderate amount | 10 (58.8) | 0 (0.0) | 9 (69.2) | 1 (50.0) |
| Numerous | 2 (11.8) | 0 (0.0) | 2 (15.4) | 0 (0.0) |
| Lymfocytic infiltrate (n=2 missing) |  |  |  |  |
| None/scarse | 0 (0.0) | 0 (0.0) | 0 (0.0) | 0 (0.0) |
| Few | 8 (19.0) | 3 (75.0) | 4 (13.3) | 1 (12.5) |
| Moderate amount | 28 (66.7) | 1 (25.0) | 20 (66.7) | 7 (87.5) |
| Numerous | 6 (14.3) | 0 (0.0) | 6 (20.0) | 0 (0.0) |
| Plasma cells (n=2 missing) |  |  |  |  |
| None/scarse | 30 (71.4) | 3 (75.0) | 21 (70.0) | 6 (75.0) |
| Few | 10 (23.8) | 1 (25.0) | 7 (23.3) | 2 (25.0) |
| Moderate amount | 1 (2.4) | 0 (0.0) | 1 (3.3) | 0 (0.0) |
| Numerous | 1 (2.4) | 0 (0.0) | 1 (3.3) | 0 (0.0) |
| Eosinophils (n=2 missing) |  |  |  |  |
| None/scarse | 23 (54.8) | 3 (75.0) | 15 (50.0) | 5 (62.5) |
| Few | 18 (42.9) | 1 (25.0) | 14 (46.7) | 3 (37.5) |
| Moderate amount | 1 (2.4) | 0 (0.0) | 1 (3.3) | 0 (0.0) |
| Numerous | 0 (0.0) | 0 (0.0) | 0 (0.0) | 0 (0.0) |
| Macrophages (n=2 missing) |  |  |  |  |
| None/scarse | 1 (2.4) | 0 (0.0) | 0 (0.0) | 1 (12.5) |
| Few | 11 (26.2) | 3 (75.0) | 5 (16.7) | 3 (37.5) |
| Moderate amount | 20 (47.6) | 0 (0.0) | 18 (60.0) | 2 (25.0) |
| Numerous | 10 (23.8) | 1 (25.0) | 7 (23.3) | 2 (25.0) |
| Thrombi (n=3 missing) | 1 (2.4) | 0 (0.0) | 1 (3.3) | 0 (0.0) |
| Pneumocyte atypia | 28 (66.7) | 1 (25.0) | 21 (70.0) | 6 (75.0) |
| Findings contributing to death (n=3 missing) |  |  |  |  |
| No pathological findings | 0 (0.0) | 0 (0.0) | 0 (0.0) | 0 (0.0) |
| Findings unlikely to contribute to death | 2 (4.9) | 1 (33.3) | 1 (3.3) | 0 (0.0) |
| Findings possibly contributing to death | 4 (9.8) | 1 (33.3) | 1 (3.3) | 2 (25.0) |
| Findings probably contributing to death | 6 (14.6) | 1 (33.3) | 3 (10.0) | 2 (25.0) |
| Findings likely to contribute to death | 29 (70.7) | 0 (0.0) | 25 (83.3) | 4 (50.0) |
| Megakaryocytes (n=2 missing) |  |  |  |  |
| None | 21 (50.0) | 1 (25.0) | 14 (46.7) | 6 (75.0) |
| One | 10 (23.8) | 1 (25.0) | 7 (23.3) | 2 (25.0) |
| Two or more | 11 (26.2) | 2 (50.0) | 9 (30.0) | 0 (0.0) |
| Vasculitits (n=2 missing) | 3 (7.1) | 0 (0.0) | 1 (3.3) | 2 (25.0) |
| CT-normal vs. CT-abnormal |  |  |  |  |
| Different | 10 (22.7) | 2 (50.0) | 7 (21.9) | 1 (12.5) |
| More or less the same | 32 (72.2) | 2 (50.0) | 23 (71.9) | 7 (87.5) |
| **Heart** |  |  |  |  |
| Fibrosis (n=2 missing) |  |  |  |  |
| None/absent | 20 (47.6) | 3 (75.0) | 15 (50.0) | 2 (25.0) |
| Mild | 13 (31.0) | 1 (25.0) | 9 (30.0) | 3 (37.5) |
| Moderate | 4 (9.5) | 0 (0.0) | 2 (6.7) | 2 (25.0) |
| Severe | 5 (11.9) | 0 (0.0) | 4 (13.3) | 1 (12.5) |
| Acute inflammation (n=2 missing) |  |  |  |  |
| None/absent | 38 (90.5) | 3 (75.0) | 29 (96.7) | 6 (75.0) |
| Mild | 3 (7.1) | (25.0) | 1 (3.3) | 1 (12.5) |
| Moderate | 0 (0.0) | 0 (0.0) | 0 (0.0) | 0 (0.0) |
| Severe | 1 (2.4) | 0 (0.0) | 0 (0.0) | 1 (12.5) |
| Chronic inflammation (n=2 missing) |  |  |  |  |
| None/absent | 33 (78.6) | 4 (100.0) | 25 (83.3) | 4 (50.0) |
| Mild | 8 (19.0) | 0 (0.0) | 5 (16.7) | 3 (37.5) |
| Moderate | 1 (2.4) | 0 (0.0) | 0 (0.0) | 1 (12.5) |
| Severe | 0 (0.0) | 0 (0.0) | 0 (0.0) | 0 (0.0) |
| CD3 (n=2 missing) |  |  |  |  |
| None/scarse | 24 (77.4) | 2 (66.7) | 18 (81.8) | 4 (66.7) |
| Few | 6 (19.4) | 1 (33.3) | 4 (18.2) | 1 (16.7) |
| Moderate amount | 1 (3.2) | 0 (0.0) | 0 (0.0) | 1 (16.7) |
| Numerous | 0 (0.0) | 0 (0.0) | 0 (0.0) | 0 (0.0) |
| Findings contributing to death (n=2 missing) |  |  |  |  |
| No pathological findings | 21 (50.0) | 3 (75.0) | 16 (53.3) | 2 (25.0) |
| Findings unlikely to contribute to death | 7 (16.7) | 1 (25.0) | 6 (20.0) | 0 (0.0) |
| Findings possibly contributing to death | 6 (14.3) | 0 (0.0) | 4 (13.3) | 2 (25.0) |
| Findings probably contributing to death | 4 (9.5) | 0 (0.0) | 2 (6.7) | 2 (25.0) |
| Findings likely to contribute to death | 4 (9.5) | 0 (0.0) | 2 (6.7) | 2 (25.0) |
| **Liver** |  |  |  |  |
| Sinus dilatation |  |  |  |  |
| None/absent | 2 (4.5) | 0 (0.0) | 1 (3.1) | 1 (12.5) |
| Mild | 14 (31.8) | 1 (25.0) | 8 (25.0) | 5 (62.5) |
| Moderate | 20 (45.5) | 3 (75.0) | 16 (50.0) | 1 (12.5) |
| Severe | 8 (18.2) | 0 (0.0) | 7 (21.9) | 1 (12.5) |
| Portal inflammation |  |  |  |  |
| None/absent | 33 (75.0) | 3 (75.0) | 23 (71.9) | 7 (87.5) |
| Mild | 11 (25.0) | 1 (25.0) | 9 (28.1) | 1 (12.5) |
| Moderate | 0 (0.0) | 0 (0.0) | 0 (0.0) | 0 (0.0) |
| Severe | 0 (0.0) | 0 (0.0) | 0 (0.0) | 0 (0.0) |
| Lobular inflammation |  |  |  |  |
| None/absent | 32 (74.4) | 3 (75.0) | 26 (83.9) | 3 (37.5) |
| Mild | 9 (20.9) | 0 (0.0) | 4 (12.9) | 5 (62.5) |
| Moderate | 2 (4.7) | 1 (25.0) | 1 (3.2) | 0 (0.0) |
| Severe | 0 (0.0) | 0 (0.0) | 0 (0.0) | 0 (0.0) |
| CD3 (n=38 missing) |  |  |  |  |
| None/scarse | 1 (16.7) | 0 (0.0) | 1 (25.0) | 0 (0.0) |
| Few | 5 (83.3) | 0 (0.0) | 3 (75.0) | 2 (100.0) |
| Moderate amount | 0 (0.0) | 0 (0.0) | 0 (0.0) | 0 (0.0) |
| Numerous | 0 (0.0) | 0 (0.0) | 0 (0.0) | 0 (0.0) |
| Steatosis |  |  |  |  |
| None/absent | 28 (63.6) | 3 (75.0) | 19 (59.4) | 6 (75.0) |
| Mild | 12 (2.3) | 1 (25.0) | 9 (28.1) | 2 (25.0) |
| Moderate | 2 (4.5) | 0 (0.0) | 2 (6.3) | 0 (0.0) |
| Severe | 2 (4.5) | 0 (0.0) | 2 (6.3) | 0 (0.0) |
| Findings contributing to death |  |  |  |  |
| No pathological findings | 4 (9.1) | 0 (0.0) | 2 (6.3) | 2 (25.0) |
| Findings unlikely to contribute to death | 1 (2.3) | 0 (0.0) | 1 (3.1) | 0 (0.0) |
| Findings possibly contributing to death | 13 (29.5) | 2 (50.0) | 8 (25.0) | 3 (37.5) |
| Findings probably contributing to death | 15 (34.1) | 1 (25.0) | 13 (40.6) | 1 (12.5) |
| Findings likely to contribute to death | 11 (25.0) | 1 (25.0) | 8 (25.0) | 2 (25.0) |
| **Kidney** |  |  |  |  |
| Acute tubular necrosis (n=2 missing) | 8 (19.0) | 2 (50.0) | 4 (13.3) | 2 (25.0) |
| Acute inflammation (n=3 missing) |  |  |  |  |
| None/absent | 36 (87.8) | 3 (75.0) | 27 (93.1) | 6 (75.0) |
| Mild | 3 (7.1) | 1 (25.0) | 1 (3.4) | 1 (12.5) |
| Moderate | 1 (2.4) | 0 (0.0) | 1 (3.4) | 0 (0.0) |
| Severe | 1 (2.4) | 0 (0.0) | 0 (0.0) | 1 (12.5) |
| Chronic inflammation (n=3 missing) |  |  |  |  |
| None/absent | 12 (29.3) | 2 (50.0) | 8 (27.6) | 2 (25.0) |
| Mild | 20 (48.8) | 0 (0.0) | 15 (51.7) | 5 (62.5) |
| Moderate | 8 (19.5) | 2 (50.0) | 5 (17.2) | 1 (12.5) |
| Severe | 1 (2.4) | 0 (0.0) | 1 (3.4) | 0 (0.0) |
| Findings contributing to death (n=2 missing) |  |  |  |  |
| No pathological findings | 4 (9.5) | 0 (0.0) | 4 (13.3) | 0 (0.0) |
| Findings unlikely to contribute to death | 22 (52.4) | 2 (50.0) | 15 (50.0) | 5 (62.5) |
| Findings possibly contributing to death | 9 (21.4) | 1 (25.0) | 7 (23.3) | 1 (12.5) |
| Findings probably contributing to death | 4 (9.5) | 1 (25.0) | 3 (10.0) | 0 (0.0) |
| Findings likely to contribute to death | 3 (7.1) | 0 (0.0) | 1 (3.3) | 2 (25.0) |

**Supplementary Table 3.** Relevant pathogens identified in cultures of post-mortem lung tissue.

| Pathogen |  |
| --- | --- |
| *Escherichia coli* | 6 |
| *Escherichia coli (ESBL producing)* | 1 |
| *Klebsiella pneumoniae* | 1 |
| *Klebsiella pneumonia (ESBL producing)* | 1 |
| *Klebsiella oxytoca* | 2 |
| *Klebsiella aerogenes* | 1 |
| *Proteus mirabilis* | 1 |
| *Enterobacter cloacae complex* | 1 |
| *Citrobacter freundii* | 1 |
| *Enterococcus faecium* | 2 |
| *Enterococcus faecalis* | 1 |
| *Streptococcus agalacticae* | 1 |
| *Staphylococcus aureus (methicillin susceptible)* | 1 |
| *Staphylococcus aureus (methicillin resistant)* | 1 |
| *Aspergillus fumigatus* | 1 |

**Supplementary Table 4.** All diagnoses (clinical and MIA), COD, changes and contributing disciplines. Overall, MIA provided new insights in 37/48 (77.0%) patients when compared to clinical diagnoses. In total, 44 diagnosis were changed based on MIA results: 17 in COD (‘COD revealed’ (n=7), ‘CCOD rejected’ (n=2), ‘CCOD made more specific’ (n=3), ‘CCOD made more certain’ (n=2), ‘making clinical contributing diagnosis the COD’ (n=2) and while one CCOD was made less certain.) and 27 in contributing diagnoses (‘contributing diagnosis revealed’ (n=10), ‘contributing diagnosis dismissed’ (n=8), ‘clinical diagnosis made more specific’ (n=4), ‘clinical diagnosis made more certain’ (n=5)).

|  |  |  | **Overall new insight?** | **Contributed what?** | **Contribution and rank of discipline?** | | | |
| --- | --- | --- | --- | --- | --- | --- | --- | --- |
|  |  |  |  |  | **CLIN** | **RAD** | **MIC** | **PATH** |
| 1 | Clinical COD | Small cell lung carcinoma with metastasis | Yes | Reveal contributing diagniosis | No | 1 | No | No |
|  | Contibuting Diagnosis | COVID-19 - mild illness |  |  |  |  |  |  |
|  | MIA COD | Small cell lung carcinoma with metastasis |  |  |  |  |  |  |
|  | Contibuting Diagnosis | COVID-19 - mild illness |  |  |  |  |  |  |
|  | Contibuting Diagnosis | Pancreatitis eci |  |  |  |  |  |  |
| 2 | Clinical COD | Hemorrhagic and semirecent ischemic cerebrovascular accident | Yes | Reveal contributing diagnosis | No | No | 2 | 1 |
|  | Contributing Diagnosis | Depression with refusal of food and medical interventions | | | | |  |  |
|  | Contributing Diagnosis | COVID-19 - mild illness |  |  |  |  |  |  |
|  | MIA COD | Hemorrhagic and semirecent ischemic cerebrovascular accident |  |  |  |  |  |  |
|  | Contibuting Diagnosis | Depression with refusal of food and medical interventions |  |  |  |  |  |  |
|  | Contibuting Diagnosis | COVID-19 - mild illness |  |  |  |  |  |  |
|  | Contibuting Diagnosis | Bacterial pneumonia |  |  |  |  |  |  |
| 3 | Clinical COD | Suspected pneumonia (left) | Yes | More certain COD, More specific contributing diagnosis | No | No | 2 | 1 |
|  | Contributing Diagnosis | AKI |  |  |  |  |  |  |
|  | Contributing Diagnosis | Suspected NSTEMI type 2 |  |  |  |  |  |  |
|  | MIA COD | Heart failure |  |  |  |  |  |  |
|  | Contibuting Diagnosis | COVID-19 infectio, (non-pneumonia) |  |  |  |  |  |  |
|  | Contibuting Diagnosis | Bacterial pneumonia |  |  |  |  |  |  |
| 4 | Clinical COD | Small intestine ischemia | Yes | Make contributing diagnosis more specific (COVID) | No | No | No | 1 |
|  | Contributing Diagnosis | De novo atrial fibrillation | |  |  |  |  |  |
|  | Contributing Diagnosis | Acute on CKI |  |  |  |  |  |  |
|  | Contributing Diagnosis | COVID-19 infection |  |  |  |  |  |  |
|  | MIA COD | MOF as a result of small intestine ischemia secondary to emboly because of AF and cardiomyopathy |  |  |  |  |  |  |
|  | Contibuting Diagnosis | COVID-19 mild pneumonia |  |  |  |  |  |  |
| 5 | Clinical COD | Rabdomyolysis with subsequent MOF including renal failure with dialysis | No | - | - | - | - | - |
|  | Contibuting Diagnosis | COVID-19 severe pneumonia clinically improving |  |  |  |  |  |  |
|  | MIA COD | Rabdomyolysis ECI with subsequent MOF including renal failure with dialysis |  |  |  |  |  |  |
|  | Contibuting Diagnosis | COVID severe pneumonia |  |  |  |  |  |  |
| 6 | Clinical COD | COVID-19 severe pneumonia | Yes | Reveal COD | No | No | No | 1 |
|  | Contibuting Diagnosis | Massive pulmonary embolism | | |  |  |  |  |
|  | Contributing Diagnosis | Hepatitis eci |  |  |  |  |  |  |
|  | MIA COD | Massive pulmonary embolism |  |  |  |  |  |  |
|  | Contibuting Diagnosis | COVID-19 severe pneumonia | | |  |  |  |  |
|  | Contibuting Diagnosis | Hepatitis due to right sided heart failure | | |  |  |  |  |
| 7 | Clinical COD | Intracerebral bleeding | Yes | Make contributing diagnosis more specific | 2 | No | No | 1 |
|  | Contibuting Diagnosis | Renal failure eci leading to dialysis | | |  |  |  |  |
|  | Contributing Diagnosis | COVID-19 severe pneumonia |  |  |  |  |  |  |
|  | MIA COD | Intracerebral bleeding |  |  |  |  |  |  |
|  | Contibuting Diagnosis | Renal failure due to ATN leading to dialysis |  |  |  |  |  |  |
|  | Contibuting Diagnosis | COVID severe pneumonia |  |  |  |  |  |  |
|  | Contibuting Diagnosis | Steatohepatitis |  |  |  |  |  |  |
| 8 | Clinical COD | COVID-19 severe pneumonia | Yes | Reveal contributing diagnoses | 2 | 1 | No | 3 |
|  | Contibuting Diagnosis | Acute on chronic renal failure |  |  |  |  |  |  |
|  | MIA COD | COVID-19 severe pneumonia |  |  |  |  |  |  |
|  | Contibuting Diagnosis | No renal biopsy performed | |  |  |  |  |  |
|  | Contibuting Diagnosis | Left-and right sided heart failure |  |  |  |  |  |  |
|  | Contibuting Diagnosis | Subileus |  |  |  |  |  |  |
| 9 | Clinical COD | Probable invasive Aspergillus fumigatus pulmonary infection | Yes | Make contributing diagnosis less certain | 1 | 1 | 1 | 1 |
|  | Contibuting Diagnosis | COVID-19 severe pneumonia | | |  |  |  |  |
|  | Contributing Diagnosis | Cerebral B-cell lymphoma |  |  |  |  |  |  |
|  | MIA COD | COVID-19 severe pneumonia |  |  |  |  |  |  |
|  | Contibuting Diagnosis | Probable invasive Aspergillus fumigatus pulmonary infection |  |  |  |  |  |  |
|  | Contibuting Diagnosis | Cerebral B-cell lymphoma |  |  |  |  |  |  |
| 10 | Clinical COD | COVID-19 severe pneumonia | Yes | Dismiss contributing diagnosis | No | No | No | 1 |
|  | Contibuting Diagnosis | Bacterial COPD excacerbation |  |  |  |  |  |  |
|  | MIA COD | COVID-19 severe pneumonia |  |  |  |  |  |  |
| 11 | Clinical COD | Intracranial bleeding with subdural hematoma | No | - | - | - | - | - |
|  | Contibuting Diagnosis | COVID-19 severe pneumonia |  |  |  |  |  |  |
|  | MIA COD | Intracranial bleeding with subdural hematoma |  |  |  |  |  |  |
|  | Contibuting Diagnosis | COVID-19 severe pneumonia |  |  |  |  |  |  |
| 12 | Clinical COD | COVID-19 pneumonia | Yes | Reveal contributing diagnosis | No | 2 | No | 1 |
|  | Contributing Diagnosis | Hospital acquired pneumonia |  |  |  |  |  |  |
|  | MIA COD | COVID-19 severe pneumonia |  |  |  |  |  |  |
|  | Contibuting Diagnosis | Left- and right sides heart failure |  |  |  |  |  |  |
| 13 | Clinical COD | COVID-19 Pneumonia | Yes | Dismiss contributing diagnosis | No | No | 2 | 1 |
|  | Contributing Diagnosis | Post-anoxic encephalopathy after out-of-hospital cardiac arrest | | | | |  |  |
|  | Contributing Diagnosis | Hospital acquired pneumonia |  |  |  |  |  |  |
|  | MIA COD | COVID-19 severe pneumonia |  |  |  |  |  |  |
|  | Contibuting Diagnosis | Post-anoxic encephalopathy after out-of-hospital cardiac arrest |  |  |  |  |  |  |
| 14 | Contibuting Diagnosis | CMV reactivation | Yes | Make more certain contributing diagnosis (pancreatitis), reveal contributing diagnosis (myocarditis) | No | No | No | 1 |
|  | Clinical COD | COVID-19 pneumonia with respiratory detoriation | | | |  |  |  |
|  | Contributing Diagnosis | Acute on chronic kidney insufficiency | | |  |  |  |  |
|  | Contributing Diagnosis | Atrial fibrillation with response and no hemodynamic repercussion | | | | |  |  |
|  | Contributing Diagnosis | Pancreatitis |  |  |  |  |  |  |
|  | Contributing Diagnosis | Insuline dependent diabetes mellitus |  |  |  |  |  |  |
|  | MIA COD | COVID-19 severe pneumonia + possible myositis |  |  |  |  |  |  |
|  | Contibuting Diagnosis | Semirecent/chronic pancreatitis, most likely biliary |  |  |  |  |  |  |
|  | Contibuting Diagnosis | B-CLL |  |  |  |  |  |  |
| 15 | Clinical COD | COVID-19 infection | Yes | Reveal COD | No | 2 | No | 1 |
|  | Contributing Diagnosis | Uncomplicated cystitis at admission | | |  |  |  |  |
|  | Contributing Diagnosis | Chronic kidney insufficiency | | |  |  |  |  |
|  | Contributing Diagnosis | Ferriprive blood count |  |  |  |  |  |  |
|  | MIA COD | Heart failure |  |  |  |  |  |  |
|  | Contibuting Diagnosis | COVID-19 infection with possible pneumonia |  |  |  |  |  |  |
| 16 | Clinical COD | COVID-19 pneumonia | Yes | Reveal contributing diagnosis | No | No | 2 | 1 |
|  | Contributing Diagnosis | Delirium |  |  |  |  |  |  |
|  | Contributing Diagnosis | Deshydratation | |  |  |  |  |  |
|  | Contributing Diagnosis | Atrial fibrillation | |  |  |  |  |  |
|  | Contributing Diagnosis | Rhabdomyolysis |  |  |  |  |  |  |
|  | MIA COD | COVID-19 severe pneumonia |  |  |  |  |  |  |
|  | Contibuting Diagnosis | Bacterial pneumonia |  |  |  |  |  |  |
| 17 | Clinical COD | COVID-19 pneumonia | Yes | More specific COD | No | 3 | 2 | 1 |
|  | Contributing Diagnosis | Multi-organfailure with shock |  |  |  |  |  |  |
|  | MIA COD | Pneumosepsis with septic shock because of *E. coli* |  |  |  |  |  |  |
|  | Contibuting Diagnosis | COVID-19 severe pneumonia |  |  |  |  |  |  |
| 18 | Clinical COD | COVID-19 pneumonia | No | - | - | - | - | - |
|  | Contributing Diagnosis | Neutropenic fever with MDS |  |  |  |  |  |  |
|  | MIA COD | COVID-19 severe pneumonia |  |  |  |  |  |  |
|  | Contibuting Diagnosis | MDS |  |  |  |  |  |  |
| 19 | Clinical COD | COVID-19 pneumonia with ARDS en multi-organfailure | No | - | - | - | - | - |
|  | Contributing Diagnosis | Recent fall with rib fractures | | |  |  |  |  |
|  | Contributing Diagnosis | Acute on CKI |  |  |  |  |  |  |
|  | MIA COD | COVID-19 severe pneumonia |  |  |  |  |  |  |
| 20 | Clinical COD | COVID-19 pneumonia with hypoxic respiratory failure | Yes | Make CCOD less certain | No | 1 | No | 2 |
|  | Contributing Diagnosis | Acute on CKI |  |  |  |  |  |  |
|  | Contributing Diagnosis | Old hemorragic CVA |  |  |  |  |  |  |
|  | MIA COD | Unknown |  |  |  |  |  |  |
|  | Contibuting Diagnosis | COVID-19 mild pneumonia |  |  |  |  |  |  |
| 21 | Clinical COD | COVID-19 pneumonia with high oxygen requirements | Yes | Reveal contributing diagnosis (liver necrosis), Dismiss contributing diagnosis (*E. coli* sepsis) | 2 | No | No | 1 |
|  | Contributing Diagnosis | HFrEF |  |  |  |  |  |  |
|  | Contributing Diagnosis | Permanent atrial fibrillation | | |  |  |  |  |
|  | Contributing Diagnosis | Peripheral vascular disease | |  |  |  |  |  |
|  | Contributing Diagnosis | Diabetes mellitus type II (DMII) | | |  |  |  |  |
|  | Contributing Diagnosis | CKI |  |  |  |  |  |  |
|  | Contributing Diagnosis | Recent cholangitis, now *E. coli* sepsis |  |  |  |  |  |  |
|  | MIA COD | COVID-19 severe pneumonia |  |  |  |  |  |  |
|  | Contibuting Diagnosis | Acute cardial ischemia |  |  |  |  |  |  |
|  | Contibuting Diagnosis | Spotty liver necrosis eci |  |  |  |  |  |  |
| 22 | Clinical COD | COVID-19 pneumonia | Yes | Dismiss contributing diagnosis (catheter sepsis) | 1 | No | No | No |
|  | Contributing Diagnosis | Catheter sepsis |  |  |  |  |  |  |
|  | MIA COD | COVID-19 severe pneumonia |  |  |  |  |  |  |
| 23 | Clinical COD | COVID-19 Pneumonia | No | - | - | - | - | - |
|  | Contributing Diagnosis | Recovery and revalidation after CVA with left hemiparesis | | | |  |  |  |
|  | Contributing Diagnosis | Known Multiple Myeloma |  |  |  |  |  |  |
|  | MIA COD | COVID-19 severe pneumonia |  |  |  |  |  |  |
|  | Contibuting Diagnosis | Multiple Myeloma |  |  |  |  |  |  |
|  | Contibuting Diagnosis | semi recent Ischemic CVA |  |  |  |  |  |  |
| 24 | Clinical COD | COVID-19 with rapid detoriation | Yes | Make COD more certain, reveal new contributing diagnosis | No | 2 | No | 1 |
|  | MIA COD | COVID-19 severe pneumonia |  |  |  |  |  |  |
|  | Contibuting Diagnosis | Heart failure |  |  |  |  |  |  |
| 25 | Clinical COD | COVID-19 pneumonia | Yes | Change COD | N | equally | No | equally |
|  | Contributing Diagnosis | Hypochloremia | |  |  |  |  |  |
|  | Contributing Diagnosis | Atrial fibrillation |  |  |  |  |  |  |
|  | MIA COD | Severe COPD |  |  |  |  |  |  |
|  | Contibuting Diagnosis | COVID-19 mild pneumonia |  |  |  |  |  |  |
| 26 | Clinical COD | COVID-19 pneumonia | Yes | Make contributing diagnosis more certain | No | No | No | 1 |
|  | Contributing Diagnosis | Supraventricular tachycardia (AVNRT) |  |  |  |  |  |  |
|  | MIA COD | COVID-19 severe pneumonia |  |  |  |  |  |  |
|  | Contibuting Diagnosis | Liver necrosis in histopathology but no clinical hepatitis / Decompensatio cordis with normal CT-scan or secondary to COVID-19? |  |  |  |  |  |  |
| 27 | Clinical COD | COVID-19 infection | Yes | Dismiss contributing diagnosis (aspiration pneumonia) | N | 1 | N | N |
|  | Contributing Diagnosis | Aspiration pneumonia | |  |  |  |  |  |
|  | Contributing Diagnosis | End-stage Kidney failure | |  |  |  |  |  |
|  | Contributing Diagnosis | Decreased cognitive function |  |  |  |  |  |  |
|  | MIA COD | COVID-19 severe pneumonia |  |  |  |  |  |  |
| 28 | Clinical COD | COVID-19 pneumonia | No | - | - | - | - | - |
|  | Contributing Diagnosis | Ischemic heart disease, HfmrEF | | |  |  |  |  |
|  | Contributing Diagnosis | Moderate stenosis of aortica valva and atrial fibrillation | | | |  |  |  |
|  | Contributing Diagnosis | Severe CKI |  |  |  |  |  |  |
|  | Contributing Diagnosis | DM2 |  |  |  |  |  |  |
|  | Contributing Diagnosis | Anemia |  |  |  |  |  |  |
|  | Contributing Diagnosis | Problems with swallowing |  |  |  |  |  |  |
|  | MIA COD | COVID-19 severe pneumonia |  |  |  |  |  |  |
|  | Contibuting Diagnosis | heart failure secondary to stenosis of aortic valve |  |  |  |  |  |  |
| 29 | Clinical COD | COVID-19 pneumonia | Yes | Reveal COD | 3 | 1 | 4 | 2 |
|  | Contributing Diagnosis | Myelofibrosis high risk |  |  |  |  |  |  |
|  | MIA COD | Massive heart failure with pulmonary hypertension eci |  |  |  |  |  |  |
|  | Contibuting Diagnosis | COVID-pneumonia, mild |  |  |  |  |  |  |
|  | Contibuting Diagnosis | Invasieve aspergillus/K. pneumoniae? |  |  |  |  |  |  |
|  | Contibuting Diagnosis | Myelofibrose high risk |  |  |  |  |  |  |
| 30 | Clinical COD | COVID-19 pneumonia | Yes | Change COD: make COVID pneumonia as COD unlikely | No | 1 | No | 2 |
|  | Contributing Diagnosis | HFpEF |  |  |  |  |  |  |
|  | Contributing Diagnosis | Severe inflammatory blood count | | |  |  |  |  |
|  | Contributing Diagnosis | Delirium |  |  |  |  |  |  |
|  | Contributing Diagnosis | Acute on CKI |  |  |  |  |  |  |
|  | Contributing Diagnosis | Known atrial fibrillation |  |  |  |  |  |  |
|  | MIA COD | Heart failure |  |  |  |  |  |  |
|  | Contibuting Diagnosis | COVID-19 svere pneumonia |  |  |  |  |  |  |
|  | Contibuting Diagnosis | Aspiration or bacterial pneumonia |  |  |  |  |  |  |
|  | Contibuting Diagnosis | Malignancy |  |  |  |  |  |  |
| 31 | Clinical COD | Severe spondylodiscitis with epidural abscess with *S. epidermidis* | Yes | Reveal COD | No | No | 2 | 1 |
|  | Contributing Diagnosis | COVID-19 pneumonia | |  |  |  |  |  |
|  | Contributing Diagnosis | *E. coli* cystitis | |  |  |  |  |  |
|  | Contributing Diagnosis | Anemia and increasing pancytopenia | | |  |  |  |  |
|  | Contributing Diagnosis | Decubitus bump grade 2 | |  |  |  |  |  |
|  | Contributing Diagnosis | HFrEF |  |  |  |  |  |  |
|  | Contributing Diagnosis | DM2 and atrial fibrillation |  |  |  |  |  |  |
|  | MIA COD | Bacterial pneumonia (surinfection) |  |  |  |  |  |  |
|  | Contibuting Diagnosis | COVID-19 severe pneumonia |  |  |  |  |  |  |
|  | Contibuting Diagnosis | Bacterial spondylodicitis |  |  |  |  |  |  |
| 32 | Clinical COD | Suspected gastro-enteritis | Yes | Reveal COD, COVID infection as COD unlikely | No | 1 | 3 | 2 |
|  | Contributing Diagnosis | COVID-19 infection | |  |  |  |  |  |
|  | Contributing Diagnosis | Recent fall with rib fractures | | |  |  |  |  |
|  | Contributing Diagnosis | Delirium |  |  |  |  |  |  |
|  | Contributing Diagnosis | Microscopic hematuria | |  |  |  |  |  |
|  | Contributing Diagnosis | Ferriprive blood count | |  |  |  |  |  |
|  | Contributing Diagnosis | DM2 |  |  |  |  |  |  |
|  | MIA COD | Bacterial (aspiration) pneumonia |  |  |  |  |  |  |
|  | Contibuting Diagnosis | COVID-19 infection, no pneumonia |  |  |  |  |  |  |
| 33 | Clinical COD | COVID-19 pneumonia | Yes | Reveal contributing diagnosis | No | 2 | No | 1 |
|  | Contributing Diagnosis | Acute on CKI |  |  |  |  |  |  |
|  | MIA COD | COVID-19 severe pneumonia |  |  |  |  |  |  |
|  | Contibuting Diagnosis | Bacterial pneumonia |  |  |  |  |  |  |
| 34 | Clinical COD | COVID-19 pneumonia | No | - | - | - | - | - |
|  | Contributing Diagnosis | HFrEF |  |  |  |  |  |  |
|  | Contributing Diagnosis | DM2 |  |  |  |  |  |  |
|  | Contributing Diagnosis | CKI |  |  |  |  |  |  |
|  | MIA COD | COVID-19 severe pneumonia |  |  |  |  |  |  |
|  | Contibuting Diagnosis | Heart failure with knwon ischemic heart failure |  |  |  |  |  |  |
| 35 | Clinical COD | COVID-19 pneumonia | No | - | - | - | - | - |
|  | Contributing Diagnosis | CNS bacteremia without focus | | |  |  |  |  |
|  | Contributing Diagnosis | COPD Gold D |  |  |  |  |  |  |
|  | Contributing Diagnosis | Schizoaffective disorder |  |  |  |  |  |  |
|  | MIA COD | COVID-19 severe pneumonia |  |  |  |  |  |  |
|  | Contibuting Diagnosis | Pre-existant COPD Gold 4 |  |  |  |  |  |  |
| 36 | Clinical COD | Recidive hematuria - known radiocystitis | Yes | Dismiss contributing diagnosis (bacterial pneumonia) | No | No | No | 1 |
|  | Contributing Diagnosis | COVID-19 pneumonia | |  |  |  |  |  |
|  | Contributing Diagnosis | Bacterial pneumonia | |  |  |  |  |  |
|  | Contributing Diagnosis | Cystitis |  |  |  |  |  |  |
|  | Contributing Diagnosis | Known HFrEF because of aTTR amyloidosis, now cardiorenal dilemma | | | | |  |  |
|  | Contributing Diagnosis | Hyponatremia 115 mmol/L | |  |  |  |  |  |
|  | Contributing Diagnosis | Recent fall |  |  |  |  |  |  |
|  | MIA COD | Heart failure because of known cardial amyloidosis |  |  |  |  |  |  |
|  | Contibuting Diagnosis | COVID-19 severe pneumonia |  |  |  |  |  |  |
| 37 | Clinical COD | Sudden death eci | Yes | Reveal contributing diagnosis | No | 1 | No | 1 |
|  | Contibuting Diagnosis | COVID-19 severe pneumonia clinically improving |  |  |  |  |  |  |
|  | MIA COD | Sudden death eci |  |  |  |  |  |  |
|  | Contibuting Diagnosis | COVID-19 severe pneumonia clinically improving |  |  |  |  |  |  |
|  | Contibuting Diagnosis | Minor intracerebral bleeding |  |  |  |  |  |  |
|  | Contibuting Diagnosis | Sepsis |  |  |  |  |  |  |
| 38 | Clinical COD | Acute on chronic renal failure | Yes | Make COD more specific | No | No | No | 1 |
|  | Contibuting Diagnosis | COVID-19 infection, clinical uncertainty if pneumonia | | | |  |  |  |
|  | Contributing Diagnosis | Bacterial co-infection highly suspected |  |  |  |  |  |  |
|  | MIA COD | Acute on chronic renal failure due to crescentic glomerulonephritis |  |  |  |  |  |  |
|  | Contibuting Diagnosis | COVID severe pneumonia |  |  |  |  |  |  |
| 39 | Contributing Diagnosis | Para-tracheal bleeding eci while on anticoagulant therapy for deep venous thrombosis and atrial fibrillation | No | - | - | - | - | - |
|  | Contributing Diagnosis | COVID-19 severe pneumonia |  |  |  |  |  |  |
|  | MIA COD | Para-tracheal bleeding eci while on anticoagulant therapy for deep venous thrombosis and atrial fibrillation |  |  |  |  |  |  |
|  | Contibuting Diagnosis | COVID-19 severe pneumonia |  |  |  |  |  |  |
| 40 | Clinical COD | COVID-19 severe pneumonia | Yes | Make contributing diagnosis more certain | No | No | No | 1 |
|  | Contributing Diagnosis | Hospital acquired pneumonia |  |  |  |  |  |  |
|  | MIA COD | COVID-19 severe pneumonia |  |  |  |  |  |  |
|  | Contibuting Diagnosis | Hospital acquired pneumonia |  |  |  |  |  |  |
| 41 | Clinical COD | COVID-19 severe pneumonia | Yes | Make contributing diagnosis more specific, more certain contributing diagnosis | No | 2 | 1 | 2 |
|  | Contributing Diagnosis | Bronchiolits Obliterans Syndrome (BOS) induced by Rituximab | | | | |  |  |
|  | Contributing Diagnosis | B-cell non-hodgkin lymphoma stadium IVA (follicular lymphoma grade II) | | | | | |  |
|  | Contributing Diagnosis | CMV reactivation | |  |  |  |  |  |
|  | Contributing Diagnosis | Aspergillosis |  |  |  |  |  |  |
|  | Contributing Diagnosis | Colonisation with ESBL-positive *E. Coli* & *Klebsiella spp.* |  |  |  |  |  |  |
|  | MIA COD | COVID-19 severe pneumonia |  |  |  |  |  |  |
|  | Contibuting Diagnosis | Invasive fungal infection (intracranial air as a result of sinus sferoidalis fistula combined with *Aspergillus* antigen in serum) |  |  |  |  |  |  |
|  | Contibuting Diagnosis | B-cell non-hodgkin lymphoma stadium IVA (follicular lymphoma grade II) |  |  |  |  |  |  |
| 42 | Clinical COD | COVID-19 pneumonia | Yes | Change COD | No | No | No | 1 |
|  | Contributing Diagnosis | Catheter infection | |  |  |  |  |  |
|  | Contributing Diagnosis | AKI |  |  |  |  |  |  |
|  | Contributing Diagnosis | Suspected acute cholecystitis |  |  |  |  |  |  |
|  | MIA COD | Sepsis no clear focus: Pneumonia? Pyelonephritis? Cholecystitis? |  |  |  |  |  |  |
|  | Contibuting Diagnosis | COVID-19 severe pneumonia |  |  |  |  |  |  |
|  | Contibuting Diagnosis | Lung emboly |  |  |  |  |  |  |
| 43 | Clinical COD | Severe myocardial infarction (MVATS) | No | - | - | - | - | - |
|  | Contributing Diagnosis | Inflammatory syndrome | |  |  |  |  |  |
|  | Contributing Diagnosis | COVID-19 pneumonia | |  |  |  |  |  |
|  | Contributing Diagnosis | Atrial fibrillation | |  |  |  |  |  |
|  | Contributing Diagnosis | Anuria (CVVH) | |  |  |  |  |  |
|  | Contributing Diagnosis | Shockliver |  |  |  |  |  |  |
|  | Contributing Diagnosis | Multi-organ failure |  |  |  |  |  |  |
|  | MIA COD | Recidive chorda rupture with secondary sever heart failure (MI, AI) leading to cardiogenic shock with secondary shockliver |  |  |  |  |  |  |
|  | Contibuting Diagnosis | COVID-19 severe pneumonia |  |  |  |  |  |  |
| 44 | Clinical COD | COVID-19 pneumonia | No | - | - | - | - | - |
|  | Contributing Diagnosis | Bacteriala pneumonia | |  |  |  |  |  |
|  | Contributing Diagnosis | CKI |  |  |  |  |  |  |
|  | Contributing Diagnosis | Atrial fibrillation | |  |  |  |  |  |
|  | Contributing Diagnosis | Alzheimer dementia | |  |  |  |  |  |
|  | Contributing Diagnosis | Decreased intake |  |  |  |  |  |  |
|  | MIA COD | COVID-19 severe pneumonia |  |  |  |  |  |  |
|  | Contibuting Diagnosis | Bacterial pneumonia (*E. coli*) |  |  |  |  |  |  |

Legend: Numbers 1 to 4: mild to moderate disease; Numbers 5 to 36: severe to critical disease; Numbers 37 to 44: post-acute disease; Green: MIA diagnosis is certain; Yellow: MIA diagnosis is highly likely; Orange: MIA diagnosis is considered; CLIN: clinical chart review; RAD: radiological examination; MIC: microbiological examination; PATH: histopathological examination; eci: e causa ignota, B-CLL: B-cell lymphoma; MDS: Myelodysplasia; CKI: chronic kidney insufficiency; HFrEF: heart failure with reduced ejection fraction; NSTEMI: non-ST elevated myocardial infarction; ARDS: acute respiratory distress syndrome; CVA: cerebro-vasclular accident; HRpEF: heart failure with preserved ejection fraction.
